# *N*‐Functionalised Imidazoles as Stabilisers for Metal Nanoparticles in Catalysis and Anion Binding

**DOI:** 10.1002/open.202000145

**Published:** 2020-06-08

**Authors:** Christopher J. Serpell, James Cookson, Paul D. Beer

**Affiliations:** ^1^ School of Physical Sciences, Ingram Building University of Kent Canterbury CT2 7NH UK; ^2^ Johnson Matthey Technology Centre, Reading RG4 9NH; ^3^ Chemistry Research Laboratory, Department of Chemistry University of Oxford Mansfield Road Oxford OX1 3TA UK

**Keywords:** nanoparticles, ligands, anion coordination, functionalised imidazoles, sensing applications

## Abstract

Metal nanoparticles (NPs) have physicochemical properties which are distinct from both the bulk and molecular metal species, and provide opportunities in fields such as catalysis and sensing. NPs typically require protection of their surface to impede aggregation, but these coatings can also block access to the surface which would be required to take advantage of their unusual properties. Here, we show that alkyl imidazoles can stabilise Pd, Pt, Au, and Ag NPs, and delineate the limits of their synthesis. These ligands provide an intermediate level of surface protection, for which we demonstrate proof‐of‐principle in catalysis and anion binding.

## Graphical abstract

**Protecting the surface**: Imidazole ligands offer a potentially useful intermediate level of stabilisation for metal nanoparticles. We outline the parameters required for synthesis of such nanoparticles and provide two case studies for their application, in catalysis and in sensing.

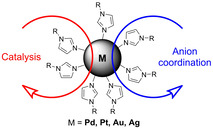

## 1 Introduction

Due to their exceptionally high surface area to volume ratio, metal nanoparticles (NPs) are natural candidates for new catalysts and sensors. The benefits of nanoparticulate systems exceed mere metal economy: the confinement of electrons to nanoscale domains can improve activity and selectivity, and offer surface‐sensitive optical properties; and a shell of stabilising ligands, polymers, or surfactants can be tailored to fine tune the interaction of the substrate with the surface.^1^, ^2^ NPs require this stabilising shell to prevent irreversible agglomeration which is catastrophic for nanoelectronic properties and surface area. Stabilisers operate either sterically (using ligands or polymers) or electrostatically, preventing the close approach of separate NPs,^3^ but this occlusion of the surface may also apply to target substrates for catalysis or sensing, resulting in reduced activity.^4^, ^5^ Conversely, a functionally active NP must have a surface which is accessible to the substrate ‐ but less robust surface coverage may also promote agglomeration. The level of stability afforded varies according to factors such as the ratio of capping agent to metal, and the magnitude of the interaction with the surface. The use of lone‐pair donating ligands which form a coordinate bond with the surface is particularly versatile because the strength of the attachment can be correlated with molecular analogues, and judicious choice of ligand can impart lability, allowing exchange with incoming ligands,^6^, ^7^ and fluidity on the surface.^8^ Such dynamic potential may promote catalytic activity. Thiols are most commonly employed since these exhibit strong interactions with noble metal surfaces; they have become the ‘default’ ligands, especially for AuNPs, since the introduction of the Brust method^9^ for synthesis, despite sulfur being well known as a catalyst poison. However, donation of electrons from other elements is entirely feasible. Nanoparticle stabilisation has been reported through nitrogen,^10^ oxygen,^11^ and phosphorous^12^ donors, and more recently carbon.^13^

The strength of the ligand‐metal interaction will play a key role in determining access of catalytic or analytic substrates to the surface. For the production of new nanoparticles for catalysis or sensing, the ideal ligand should strike a balance between strength of coordination and lability, be easily synthesised from inexpensive commercial materials, exhibit broad scope for modification, and be non‐protic. We believe that the hard/soft mismatch between nitrogen donors and noble metals could be used to achieve this, and in particular, alkyl imidazoles comply with all these requirements. To date, use of the basic imidazole nitrogen to stabilise NPs has been sparse. The first observation of NP stabilisation by alkyl imidazoles was the discovery that such ligands were present as impurities in ionic liquids, and when NPs were produced in those media, the neutral N‐donors were the true NP ligands. Indeed, intentional addition of methyl imidazole improved the uniformity and stability of AuNPs, and in PdAu alloy NPs the catalytic activity was also increased.^14^ Through incorporation of an anion binding moiety in the alkyl chain, we have employed imidazole ligands in the creation of core@shell bimetallic particles for enhancement of catalytic selectivity.^15^ The imidazole motif has been used to pre‐coordinate gold ions to the reducing biopolymer chitosan to produce polymer‐coated luminescent AuNPs which were active for catalysing hydride reductions.^16^ In a similar vein, polysiloxane microspheres were decorated with imidazole groups, and used to bind Pd which was then reduced into nanoparticles on the surface to create catalysts for hydrogenation.^17^ These studies indicate that there is further potential for exploration of catalysis by imidazole‐stabilised NPs. Beyond catalysis, there are otherwise unrelated reports of imidazole‐NPs: copper NPs produced by pulsed‐laser ablation in the presence of DNA were found to be stabilised in part by the imidazole N of purine DNA bases;^18^ replacement of one of the phenyl rings in azobenzene with an imidazole enabled binding of the photoswitching unit to AgNPs, where its switching capacity was retained;^19^ and the lability of the imidazole N−NP bond has been used in the development of sensors for sulfur mustard, a chemical warfare agent, by ligand displacement.^20^ As indicated above, in its bis‐alkylated imidazolium form, the heterocycle may provide NP stabilisation either noncovalently,^21^ or as an *N*‐heterocyclic carbene,^13^ (a stronger interaction^22^) while other imidazole‐containing ligands have been produced which provide stabilisation through a motif other than the basic nitrogen, e. g. 2‐mercapto‐1‐methylimidazole.^23^, ^24^ The related triazole heterocycle has also been used as an N‐donor to stabilise AuNPs for sensing and catalysis,^25^, ^26^, ^27^ and tetrazoles have also been explored.^28^ Supported by these positive but disparate reports, there is much unexplored potential for alkyl imidazoles as ligands for metal nanoparticles in catalysis and beyond.

Here, we report the synthesis and characterisation of gold, palladium, platinum, and silver NPs stabilised by simple alkyl imidazole ligands, defining the limiting requirements for NP stabilisation in terms of alkyl chain length, molar equivalents, and metal used. We then provide two case studies for how these NPs may be used, showing proof‐of‐principle for enhanced catalytic activity despite the presence of the ligands, and for anion coordination using functional ligands.

## 2 Results and Discussion

### 2.1 Nanoparticle Synthesis

**Scheme 1:**
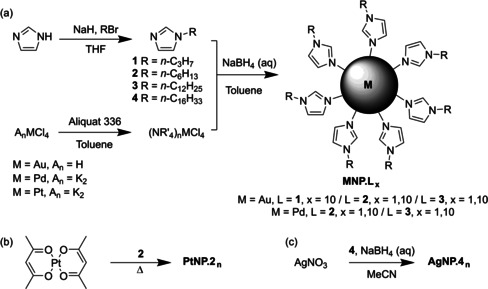
Synthetic routes towards imidazole‐stabilised NPs. (a) Brust‐style two‐phase chemical reduction. (b) Thermal degradation of platinum complexes. (c) One‐phase chemical reduction.

To make use of alkyl imidazole capping of metal NPs, stabilisation requirements and synthetic protocols needed to be established. This involved discovering the minimum required alkyl chain length and alkyl imidazole ligand : metal ratio for stabilisation, testing reaction conditions, and experimenting with a range of metals. Alkyl imidazoles were readily synthesised in excellent yield by deprotonation of imidazole with sodium hydride in THF, followed by reaction with bromoalkanes (Scheme 1a). Alkyl imidazole ligands **1**–**3** were isolated as increasingly viscous oils, while **4** was a waxy solid. NPs were then synthesised by an adaptation of the Brust method.^9^ Starting with hydrogen tetrachloroaurate, potassium tetrachloropalladate, and potassium tetrachloroplatinate, the metallate anions were extracted into toluene from water using the phase transfer agent Aliquat 336. The layers were separated, and the alkyl imidazole ligand (**1**–**3**) was added to the organic layer in molar ligand : metal ratios of 1 and 10. After cooling in an ice bath, dropwise addition of aqueous sodium borohydride followed. In case of Au, a colour change from yellow to deep purple, typical of AuNPs, was observed, whereas for Pd a black solution was obtained. When using Pt, no nanoparticle formation was observed, despite allowing the reaction to warm to room temperature, or addition of hydrochloric acid to promote the evolution of dihydrogen from the borohydride salt. Work‐up was achieved by washing with acid (to remove excess alkyl imidazoles) and water (to remove inorganic side products), followed by evaporation of the solvent, giving products **MNP.L_x_** where M is the metal used, L is the ligand (compounds **1**–**3**), and x is the number of equivalents of ligand to metal used in the reaction (not necessarily that of the product). The AuNP samples were thick oils which displayed a gold sheen despite being redispersible in organic solvents, whereas black oils were obtained for the PdNPs. Stable nanoparticles were obtained for both gold and palladium using alkyl imidazole ligands **2** and **3** in both 1 : 1 and 1 : 10 metal : ligand ratios, whereas using ligand **1**, the only stable product was **AuNP.1_10_**, indicating the smallest *n*‐propyl group was not capable of sterically stabilising PdNP formation. In an effort to obtain PtNPs, an alternative method was pursued (Scheme 1b), in which platinum acetoacetonate was dissolved in **2** and thermally decomposed, giving a colour change from orange to dark brown, characteristic of PtNPs (**PtNP.2_n_**). However, it was not possible to separate **PtNP.2_n_** from the excess **2**. Synthesis of alkyl imidazole stabilised AgNPs (starting from AgNO_3_, Scheme 1c) was attempted using ligands **2** and **3**, but gave only agglomerated bulk metal. Stable **AgNP.4_10_** could be generated using ten equivalents of hexadecylimidazole (**4**), but after reaction could not be separated from the free ligand, giving a pale pink waxy material.

**Figure 1:**
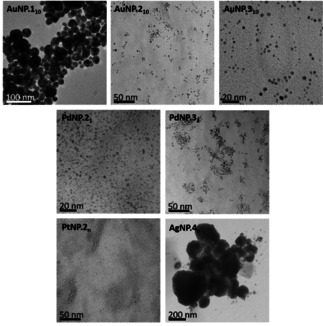
TEM images of selected alkyl imidazole‐stabilised NPs. Further images can be found in the Supporting Information.

All the NP samples were characterised by TEM (Figure 1, Table 1, Figure S1–12, Supporting Information). Large and uneven particles were seen for **AuNP.1_10_**, indicating the paucity of the stabilisation given by the short *n*‐propyl chain imidazole. Much smaller sizes and improved uniformity were seen across the board for the other AuNP samples. High magnification of **AuNP.2_1_** revealed the lattice fringes, designating the planes of metal atoms within the NP and indicating a crystalline nature. Small and uniform spherical NPs were observed for the Pd samples **PdNP.2_1_**, **PdNP.2_10_**, and **PdNP.3_10_** but irregular morphology found for **PdNP.3_1_**. Use of Pd gave smaller NPs than gold by about 1 nm in diameter, and with better monodispersity. Using either metal, the particle size was lower when ten equivalents of the ligand to metal were used, rather than equimolar ratios. This corresponds to the greater stabilised surface area that can be achieved by providing more capping agents. **PtNP.2_n_** were extremely small (<1 nm), which explains why separation from excess ligand was challenging. **AgNP.4_10_** displayed very large NPs with high dispersity. Since stable solutions were obtained of the NPs, their clustering in the TEM images is most likely a drying effect; while it is possible that agglomeration would also occur upon drying, since the NP samples could be repeatedly dried and redissolved we do not believe that this is significant. The AuNPs displayed plasmon resonance bands (PRB) in the visible region typical of the material (Table 1), whereas no PRB was observed for Pd or Pt, as is usual.^29^

**Table 1:** Characterisation data for NPs. ^a^ Toluene, 293 K. ^b^ Determined using TEM, n=number of NPs measured.

Ligand/Au molar ratio	PRB/nm^a^	Average diameter/nm^b^
**AuNP.1_10_**	532	25.5±10.1, n=165
**AuNP.2_1_**	529	2.9±1.7, n=136
**AuNP.2_10_**	520	2.8±1.1, n=118
**AuNP.3_1_**	522	3.9±1.2, n=285
**AuNP.3_10_**	520	2.3±1.0, n=398
**PdNP.2_1_**	–	1.9±0.6, n=484
**PdNP.2_10_**	–	1.6±0.9, n=130
**PdNP.3_1_**	–	2.2±0.9, n=393
**PdNP.3_10_**	–	1.9±0.2, n=32
**PtNP.2_n_**	–	<1 nm
**AgNP.4_10_**	–	25.6±36.7 nm, n=29

These studies show that stable and well‐formed NPs can be obtained using Au and Pd using alkyl imidazole ligands via the Brust method. For these, a propyl chain on the imidazole is insufficient, presumably since it does not provide sufficient steric stabilisation, but a hexyl chain is effective, as is a dodecyl chain. Ligand : metal ratios of both 1 : 1 and 10 : 1 are suitable with hexyl and dodecyl imidazoles; the higher ratio gives smaller NPs. PtNPs and AgNPs are accessible but require adapted synthetic methods and do not display useful size distributions.

### 2.2 Assessing Surface Accessibility Through Catalysis

We then proceeded to examine the degree of surface accessibility by conducting catalytic tests on the nanoparticulate metals – if the ligands prevent substrates reaching the surface, then the catalytic activity should be significantly lower than commercial catalysts, or our own samples following ligand removal. Preliminary evaluation showed that only the palladium‐based systems were catalytically active in commercially relevant hydrogenation conditions, therefore these were targeted for further investigation and comparison with ligand‐free controls.

Heterogeneous catalysts were prepared by adsorbing solutions of **PdNP.2_1_**, **PdNP.2_10_**, **PdNP.3_1_** and **PdNP.3_10_** onto activated carbon, each in projected metal loadings of both 1 wt% and 5 wt%. This was achieved by stirring a chloroform solution of NPs with carbon until the solution lost all colour (typically one hour). Filtration, washing with chloroform, and vacuum drying of the carbon suspension gave samples ready for analysis. TEM analysis of the supported particles displayed some growth in particle diameter compared to the unsupported particles; for example, **PdNP.3_10_** increased from 1.4 to 4.2 nm in average diameter – this may in part be due to a flattening effect caused by the attraction of the NP to the surface, or to partial agglomeration. The increase of particle size of precious metal nanoparticles has previously been observed and attributed to the formation of stronger bonds with defects on the surface of various supports^30^, ^31^ To test the effect of the presence or absence of ligands on the catalytic properties of the NPs, a selection of the samples were calcined in air, burning off the ligands. The appropriate temperature for this was found using thermogravimetric analysis (TGA, Figure S15, Supporting Information). For pure ligands **2** and **3**, total decomposition had occurred by 250 °C and 350 °C respectively. The main feature in the TGA plots of the Pd samples adsorbed onto carbon was the combustion of the support at 500 to 550 °C. This is unsurprising given that samples contained only 5 wt% imidazole‐stabilised NPs. Closer examination of the plots for NPs **PdNP.2_1_** and **PdNP.3_10_** reveals downward kinks in the TGA traces corresponding to the loss of ligand at 250 °C and 350 °C respectively. The total loss of mass prior to reaching 400 °C, corresponding to the ligands, was compared with the residue at 600 °C (pure metal), to estimate the surface coverage. A ligand mass% of 32.7 and 41.0 were obtained for **PdNP.2_1_** and **PdNP.3_10_** respectively, giving approximately 296 and 77 ligands per particle, or 11.2 and 6.7 ligands per nm^2^, taking into account average particle size. An estimated theoretical maximum number of ligand/nm^2^ is 12.5, indicating that with the shorter alkyl chain the ligands are closely packed, whereas with the longer chain there is greater spacing. For catalytic tests, a representative selection of NP samples were calcined at 450 °C to ensure complete ligand removal without combustion of the carbon support. Finally, the Pd samples were analysed by Inductively Coupled Plasma Emission Spectroscopy (ICP‐ES), to ascertain the precise metal loading, an important detail for preparing catalytic tests. The results show that the level of loading is uniformly lower than expected (Table 2). These losses of metal could occur through the formation of small levels of agglomerated metal which do not pass through filter paper, and through incomplete adsorption onto the carbon support.

**Table 2:** Projected and measured (ICP‐ES) loading of Pd NPs onto carbon supports.

Sample	Projected Loading	Measured Loading
**PdNP.2_1_**	1 %	0.44 %
**PdNP.2_1_**	5 %	2.27 %
**PdNP.2_10_**	1 %	0.66 %
**PdNP.2_10_**	5 %	2.82 %
**PdNP.3_1_**	1 %	0.81 %
**PdNP.3_1_**	5 %	3.45 %
**PdNP.3_10_**	1 %	0.31 %
**PdNP.3_10_**	5 %	3.45 %

**Figure 2:**
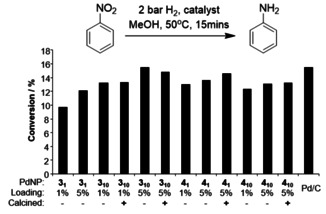
Conversion of nitrobenzene to aniline by PdNPs. 2 bar, 15 min, 50 °C, MeOH, 1 : 1000 ratio of Pd : substrate.

Nitrobenzene (NB) is widely used in organic synthesis, and as a solvent. Its reduction to aniline (An) is important both in terms of synthetic methodology and as a step in the degradation of a common pollutant.^32^ The reduction of NB to An was therefore taken as a preliminary test reaction for the catalytic activity of PdNPs. Catalytic experiments using PdNPs, varying the stabiliser chain length, metal to ligand ratio, catalyst loading, and use of calcination, were undertaken at 50 °C under 2 bar of hydrogen, the products being analysed by gas chromatography (GC). Given a 15 min stirring time, none of the reactions had reached completion, allowing a meaningful comparison of catalytic activity based upon their % conversion (Figure 2). The performance of the Pd NPs was comparable to the Pd/C standard and very minimally affected by calcination – the ratio of the % conversion for uncalcined to calcined ranged from 0.93 to 1.05. This affirms that the imidazole ligand is sufficiently labile that it does not impede access to the NP surface for catalysis, consistent with our hypothesis that the hard/soft mismatch of nitrogen donors with noble metals would provide such behaviour, and with previous results.^15^, ^20^ Although there were no clear activity trends with respect to alkyl chain length (C_6_ vs. C_12_), or ligand to metal ratio, the samples with nominally 5 wt% catalytic loading performed consistently better than those with 1 wt% coverage, despite reactions being controlled for total Pd content.

**Scheme 2:**
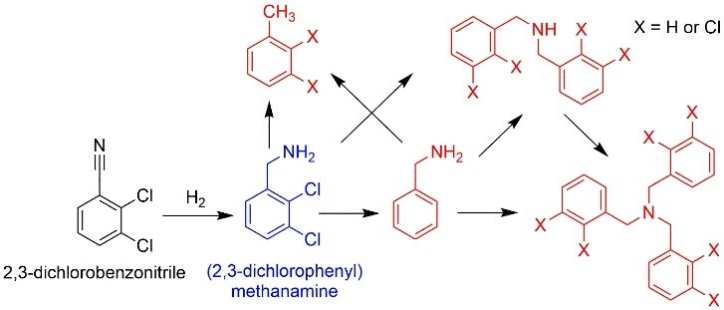
Hydrogenation pathways for 2,3‐dichlorobenzonitrile.

To test the Pd NPs on a more challenging reaction in terms of selectivity, the reduction of 2,3‐dichlorobenzonitrile was investigated. This reaction can proceed by a number of routes including dehalogenation and formation of secondary and tertiary amines (Scheme 2). The target, (2,3‐dichlorophenyl)methanamine, is an amino aryl halide, a class of compounds which are versatile synthons in organic chemistry, and for which selective reduction catalysts are highly desirable.^33^ **PdNP.3_1_** at 5 wt% loading (uncalcined, i. e. with ligands still present) were selected to be compared with the commercial Pd/C standard. The reaction was conducted in pure ethanol, and in acidic media known to promote the desired selectivity.^34^ While the product distribution was dominated by the target compound and the starting material, the presence of other products was conspicuous, notably the 3‐dechlorinated species. It is noteworthy that when compared to the Pd/C standard, a significant improvement in selectivity was observed for **PdNP.3_1_** in ethanol – mild, neutral conditions suitable for reduction of functionalised molecules (Table 3). Furthermore, the % conversion resulting from use of the alkylimidazole‐coated NPs was again close to that of the commercial Pd/C, again evidencing that access of substrates to the metal surface was not impeded (ratio of % conversion of **PdNP.3_1_** to commercial Pd/C between 0.95 and 1.16).

**Table 3:** Levels of conversion and selectivity (% of target found in product mixture) for the reduction of 2,3‐dichlorobenzonitrile to (2,3‐dichlorophenyl)methanamine catalysed by Pd NPs and Pd/C 87 L in different solvents. 4 bar, 4 hr (target 75 % conversion), 50 °C, 1 : 1000 ratio of Pd : substrate.

	Conversion	Selectivity
**PdNP.3_1_** EtOH	83.1 %	50.7 %
Pd/C EtOH	82. 8 %	38.7 %
**PdNP.3_1_** EtOH/HCl	74.9 %	82.2 %
Pd/C EtOH/HCl	64.5 %	86.0 %
**PdNP.3_1_** AcOH	73.1 %	39.1 %
Pd/C AcOH	77.1 %	34.4 %

These catalytic tests provide proof‐of‐principle for the use of alkylimidazole‐stabilised NPs as new hydrogenation catalysts, since the ligands due not diminish catalytic activity significantly, but can aid product selectivity. We believe that this is due to the hard/soft mismatch of the nitrogen donor with the noble metal surface.

### 2.3 Anion Sensing Studies Using Silver Nanoparticles Stabilised by a Bis‐Imidazolium‐Imidazole Ligand

We then decided to examine the possibility of non‐covalently binding a second metal to the surface prior to reduction, to generate core@shell nanoparticles. This templation‐via‐anion‐coordination strategy has been used to produce Au/Pd core@shell NPs which showed improved reduction of chloronitroaniline without dehalogenation compared to monometallic or unstructured bimetallic species,^15^ but the system was not universally applicable. Here, we decided to investigate the use of a more potent anion‐host based upon charge‐assisted hydrogen bonding through use of a pre‐macrocycle **5** 35 which binds chloride in the highly competitive DMSO‐d_6_ (K=164 (6) mol^−1^ dm^3^), while the cyclised version is stronger yet at K=420 (23) mol^−1^ dm^3^; coordination to the NP surface could be considered a type of cyclisation and thus result in similar strength. An increase in anion binding affinity could improve the synthetic efficiency of the core@shell structure preparation since it would prevent formation of monometallic NPs during the second reduction step.

**Scheme 3:**
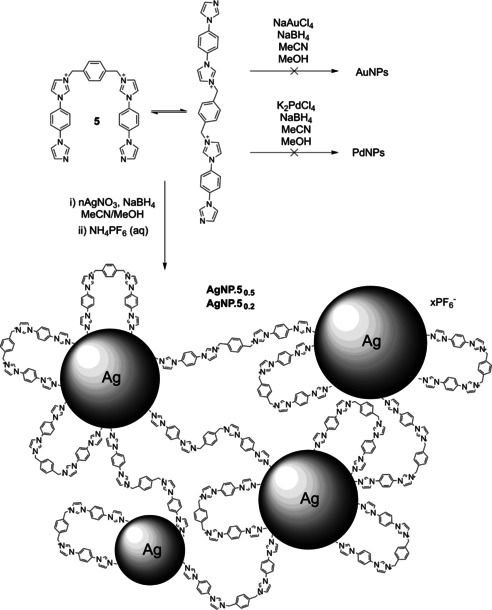
Synthesis of AgNPs using bidentate imidazolium‐imidazole ligands, and proposed structure of aggregates.

**Figure 3:**
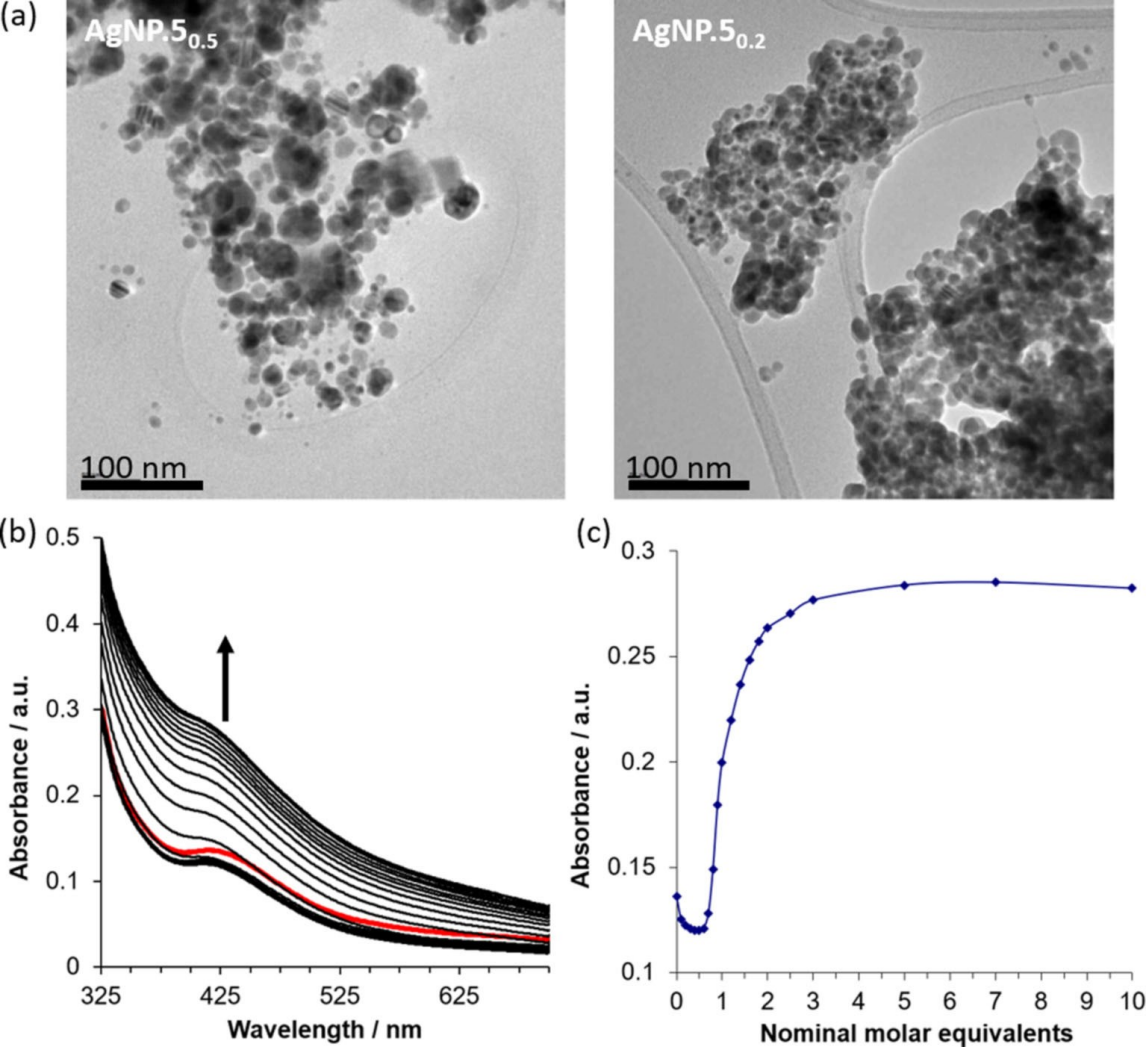
Characterisation of AgNPs. (a) TEM images (more can be found in the SI). (b) Change in UV‐visible spectrum of **AgNP.5_0.5_** upon addition of 1–10 equivalents of TBA chloride in 1 : 1 MeCN : H_2_O (298 K). (c) Change in intensity of UV‐visible spectrum at 413 nm according to apparent equivalents of chloride added.

Borohydride reductions were attempted in acetonitrile (required for solubility of **5**) using NaAuCl_4_, K_2_PdCl_4_, or AgNO_3_ as metal salt precursors, with both 0.5 and 0.2 equivalents of the ligand (Scheme 3). Agglomerated bulk metal was obtained from the Au salt, while addition of the Pd salt caused precipitation of a highly insoluble material (presumably **5**.PdCl_4_) prior to reduction. After dispersing that material as a suspension, addition of methanolic sodium borohydride generated only insoluble agglomerated products. The outcome was different with the Ag salt, the solution of which turned yellow and then dark brown, giving stable NPs as the sole product in the 1 : 2 ligand : metal ratio (**AgNP.5_0.5_**), some agglomerated material at 1 : 5 ratio (**AgNP.5_0.2_**). Filtration was performed and the solvent was removed in vacuo, the residue being then triturated with saturated aqueous NH_4_PF_6_, to give **AgNP.5_0.5_** and **AgNP.5_0.2_** as their non‐coordinating hexafluorophosphate salts. The samples were characterised by UV‐visible spectroscopy, powder X‐ray diffraction (PXRD), and TEM analysis. PXRD revealed that while the major component of **AgNP.5_0.2_** was identical to bulk silver, this metallic form was a much smaller part of **AgNP.5_0.5_**. Scherrer analysis of the (220) reflection gave diameters of 10.3 and 8.5 nm for **AgNP.5_0.5_** and **AgNP.5_0.2_** respectively. UV‐visible spectroscopy revealed plasmon resonance bands at 419 and 413 nm for **AgNP.5_0.5_** and **AgNP.5_0.2_** respectively, both of which are typical for Ag NPs of approximately 10 nm. This size analysis was confirmed by TEM experiments. (Figure 3a, Fig S13–S14) which gave diameters of 11.1 nm (±1.8, n=85) and 7.5 nm (±1.6, n=154) for **AgNP.5_0.5_** and **AgNP.5_0.2_** respectively. Compared to other NP samples, it can be seen from the TEM images that the AgNPs form aggregates rather than being dispersed evenly. Given the dense nature of the aggregates and their presence throughout the TEM sample, we do not believe that this is related to drying. However, unlike most aggregated NPs, we found that the AgNPs were readily redispersible. This is likely to be due to the bidentate nature of **5**, and its lack of preorganisation – the imidazole nitrogens may equally coordinate one or two NPs, producing an interlinked network as postulated in Scheme 3.

Anion binding of the AgNP network was examined via the effect of the addition of anions upon the plasmon resonance band (PRB) of **AgNP.5_0.5_** using UV‐visible spectroscopy (Figure 3b,c). Experiments were undertaken titrating **AgNP.5_0.5_** with tetrabutylammonium (TBA) salts with the aim of establishing surface binding before creating core@shell NP systems. A particular difficulty encountered when performing titrations using NPs is that the exact receptor concentration is difficult to ascertain. To determine this, with the prior knowledge that in acetoitrile, addition of one equivalent of TBA Cl to **5** caused precipitation, we titrated **AgNP.5_0.5_** with TBA Cl in same conditions. The point at which precipitation was observed was then calibrated to the one equivalent mark. To prevent precipitation in deeper analysis of the anion binding properties of **AgNP.5_0.5_**, titrations were subsequently performed in 1 : 1 acetonitrile/water. Addition of TBA Cl caused an overall blue shift from 413 nm to ca. 405 nm for the PRB, which was accompanied by non‐linear changes in absorbance intensity – an initial decrease was followed at the one equivalent mark by a large increase, which levelled off rapidly (Figure 3b,c). The retention of the PRB indicates that the chloride ions are not dissolving the metal, as can occur in weakly stabilised AgNPs due to the formation of an Ag_2_O layer.^36^ Titrations curves of this shape are difficult to interpret, but two important facts can be extracted. Firstly, the clearly non‐linear change in intensity means that the halide anions are interacting with the NPs as well as the ligands. Secondly, since there is a strong response at the nominal one equivalent mark, it validates the stoichiometric designation from the acetonitrile titration. A distinctly different effect was seen upon the addition of TBA AuCl_4_. The PRB was quenched almost immediately, indicating a significant interaction or dissolution of the Ag, after which the spectrum of the added salt dominated, giving a linear increase in absorbance. The superimposition of spectra also occurred when TBA_2_ Pd_2_Cl_6_ was used. In this case, the absorption spectrum of the salt coincided with the PRB, so it was impossible to determine if the PRB was similarly quenched. Attempts were then made to synthesise Ag@Au and Ag@Pd NPs by combining **AgNP.5_0.5_** with ‘one equivalent’ of TBA salts of AuCl_4_^−^ or Pd_2_Cl_6_^2−^ in acetonitrile, followed by addition of methanolic sodium borohydride. In both cases this produced a dark precipitate which could not be redissolved, indicative of agglomerated metal. The formation of core@shell nanoparticles has therefore not been successful in this case, either due to the nature of the nanoparticle network, or the reduced stabilisation power of the ligand after reduction of the second metal. Nonetheless, since the spectral changes due to chloride anion addition are in the visible region, the network material obtained could act as a potential optical sensor for chloride or other anions which do not absorb light in the range of the PRB.

## 3 Conclusion

We have outlined routes to obtain Au, Pd, Pt, and Ag NPs stabilised through the basic nitrogen atoms of alkyl imidazole molecules. The Brust method can be readily adapted for Au and Pd systems, but Ag and Pt NPs were better produced by more dramatic adaptations. An *n*‐propyl chain on the imidazole provides insufficient stabilisation for high‐quality NPs, but *n*‐hexyl yields robust protection of the NPs. All alkyl imidazole systems produced NPs which were readily redispersible in a range of organic solvents (introduction of polar groups in place of alkyl ligands is also possible, permitting tuning of solubility^15^, ^20^). The ligands appear to offer minimal barrier to catalysis, a feature which we attribute to the hard‐soft mismatch of nitrogen ligands with the metals chosen here. In particular, selectivity was improved, with lower levels of dehalogenation compared to commercial Pd/C catalysts. We have also shown the use of a functional imidazole ligand can produce nanoparticle networks which report chloride binding in aqueous solvents through a non‐linear response of their plasmon resonance band. Functionalised imidazoles are versatile ligands for metal nanoparticles and their finely tuned surface interaction have potential to lead to new advances in catalysis and sensing.

## Experimental Section

All experimental details, synthetic procedures, further characterisation of NPs (TEM, TGA, PXRD), and anion binding studies can be found in the Supporting Information.

## Conflict of interest

The authors declare no conflict of interest.

## Supporting information

As a service to our authors and readers, this journal provides supporting information supplied by the authors. Such materials are peer reviewed and may be re‐organized for online delivery, but are not copy‐edited or typeset. Technical support issues arising from supporting information (other than missing files) should be addressed to the authors.

Supplementary
