# Supplementary material for: N‐Functionalised Imidazoles as Stabilisers for Metal Nanoparticles in Catalysis and Anion Binding
Source: ChemistryOpen. 2020 Jun 8;9(6):683–90. doi: 10.1002/open.202000145 (PMC7280736; doi:10.1002/open.202000145)
Supplement: Supplementary file 1 — Supplementary [file OPEN-9-683-s001.pdf]

ChemistryOpen

Supporting Information

N-Functionalised Imidazoles as Stabilisers for Metal Nanoparticles in Catalysis and Anion Binding

Christopher J. Serpell,* James Cookson, and Paul D. Beer*© 2020 The Authors. Published by Wiley-VCH Verlag GmbH & Co. KGaA. This is an open access article under the terms of the Creative Commons Attribution License, which permits use, distribution and reproduction in any medium, provided the original work is properly cited.Functional Supramolecular Systems

Supplementary Information

Contents

Materials, instrumentation, and general considerations	2
Synthesis and characterisation	3
Thermogravimetric analysis	18
Powder X-ray diffraction	19
UV-visible titrations	20
References	20

Materials, instrumentation, and general considerations

All solvents and starting materials were purchased from Fisher Scientific, Sigma-Aldrich, Arcos Organics and Alfa Aesar. Norit GSX activated carbon was used to support NPs. Materials were used as received from the supplier without any further purification methods carried out unless otherwise stated.

Routine ^{1}H and ^{13}C NMR spectra were recorded on a Varian Mercury VX300 spectrometer. All ^{13}C spectra were proton decoupled.

Routine mass spectrometry was performed on a Bruker microTOF (ESI).

UV-vis spectra were recorded on a PG Instruments T60U spectrometer or Shimadzu UV-2401PC UV-Vis photospectrometer. Samples were prepared in a 3 mL quartz cuvette and concentrations were adjusted to suit the absorption range of the instrument.

ICP-ES analyses were undertaken using a PerkinElmer Optima 3300RL instrument. Samples were prepared by Na_2O_2 infusion in a zirconium crucible and compared against standard gravimetrically determined solutions.

TEM was conducted using a Tecnai F20 Transmission Electron Microscope with a voltage of 200 kV, and C2 aperture of 30 & 50 m, in bright field (BF) and STEM (HAADF) mode, and performing *in situ* EDX analysis. A small portion of the sample was crushed and then dusted onto a holey carbon film on a TEM grid (Cu).

Thermo-gravimetric analysis was performed using a PerkinElmer Diamond TG/DTA.

Calcination was performed using a Carbolite RHF 1600 furnace.

Catalytic hydrogenations were performed in a Baskerville multivessel reactor equipped with a Buchi gas flow and pressure controller. Within each reaction cell 5.0 ml of a 0.5 mol dm^{-3} solution of the substrate in ethanol with 1,4-dioxane (internal standard for GC) was used. The carbon-supported catalyst was added in the ratio 1:1000 molar ratio of Pd to substrate, making use of the ICP-ES data for bulk composition. JM catalyst 87L was used as the commercial Pd/C standard. The reactions were run at 50 °C under 3 bar of H_2, after which the reaction mixture was extracted into CH_2Cl_2 and 10 M NaOH (aq) in order to neutralise any aniline salts formed. The composition of the resulting organic phase was analysed by gas chromatography using a PerkinElmer Autosample XL Gas chromatograph fitted with a 30m PE-5MS column. Quantification was achieved by comparison to known standard solutions. The GC was connected to a PerkinElmer TurboMass mass spectrometer to allow for determination of unknown peaks.

Powder X-ray diffraction data were recorded on a Bruker AXS D-500 using Ni filtered Cu $K\alpha$ radiation. The diffraction pattern was scanned from 20 to 100° 2θ at ambient temperature. The data were analysed using Bruker AXS Diffrac Plus, and the crystallite size was measured by the Scherrer method[1] (with the Scherrer constant K = 0.9). Profile fitting (Topas 1, Bruker-AXS) was necessary in order to deconvolute fully all peaks (i.e. standards and experimental peaks).

Synthesis and Characterisation

1-Hexyl-1*H*-imidazole (2).

Sodium hydride (60 % dispersion in mineral oil, 3.15 g, 78.8 mmol) was suspended in 100 ml dry THF under nitrogen at 0 °C. Imidazole (4.47 g, 65.7 mmol) was dissolved in 50 ml dry THF and added dropwise. After gas evolution stopped, 1-bromohexane (10.85 g, 65.7 mmol) in 50 ml dry THF was also added dropwise. The resultant mixture was then stirred at room temperature overnight. After careful addition of a few drops of water to quench the hydride, the organic solvent was removed and diethyl ether (75 ml) was added. This solution was washed three times with water (50 ml), and the combined aqueous layers were re-extracted with diethyl ether (50 ml). The collected organic layers were then dried over $MgSO_4$ and filtered. Solvent removal gave the product as a yellow oil (9.74 g, 97 %). ^{1}H NMR (300 MHz, $CDCl_3$) δ (ppm) 7.47 (1H, s, Im*H*), 7.06 (1H, s, Im*H*), 6.91 (1H, s, Imr*H*), 3.93 (2H, t, ^{3}J = 7.2 Hz, NC*H*$_2$), 1.77, (2H, m, NCH_2C*H*$_2$), 1.29 (6H, m, -(C*H*$_2$)$_3$-), 0.88 (6H, t, ^{3}J = 6.7 Hz, CH_2C*H*$_3$); ESMS: *m/z* calc. for $[M + H]^+$ 153.13, found 153.12.

1-Propyl-1*H*-imidazole (1).

Preparation as for **2**, using imidazole (0.25 g, 3.67 mmol), 1-bromopropane (0.44 g, 3.67 mmol) and NaH (60% dispersion in mineral oil, 0.15 g, 3.8 mmol), giving **1** as a pale yellow oil (0.40 g, 100 %). ^{1}H NMR (300 MHz, $CDCl_3$) δ (ppm) 7.45 (1H, s, Im*H*), 7.04 (1H, s, Im*H*), 6.89 (1H, s, Im*H*), 3.88 (2H, t, ^{3}J = 7.0 Hz, C*H*$_2$), 1.79 (2H, tq, ^{3}J = 7.0, 7.3 Hz, C*H*$_2$), 0.91 (3H, t, ^{3}J = 7.3 Hz, C*H*$_3$); ESMS: *m/z* calc. for $[M + H]^+$ 111.09, found 111.09.

1-Dodecyl-1*H*-imidazole (3).

Preparation as for **2**, using imidazole (1.50 g, 0.022 mol), 1-bromododecane (5.45 g, 0.22 mol) and NaH (60% dispersion in mineral oil, 0.88 g, 0.022 mol), giving **116** as a pale yellow oil (5.08 g, 98 %). ^{1}H NMR (300 MHz, $CDCl_3$) δ (ppm) 7.43 (1H, s, Im*H*), 7.02 (1H, s, Im*H*), 6.87 (1H, s, Im*H*), 3.89 (2H, t, ^{3}J = 7.2 Hz, C*H*$_2$), 1.74 (2H, m, C*H*$_2$), 1.21 (18H, m, C*H*$_2$), 0.85 (3H, t, ^{3}J = 7.0 Hz, C*H*$_3$); ESMS: *m/z* calc. for $[M + H]^+$ 237.23, found 237.20.

1-Hexadecyl-1*H*-imidazole (4).

Preparation as for **2**, using imidazole (0.25 g, 3.67 mmol), 1-bromohexadecane (1.11 g, 3.67 mmol) and NaH (60% dispersion in mineral oil, 0.15 g, 3.67 mmol), giving **117** as a cream-coloured waxy solid (1.11 g, 100 %). ^{1}H NMR (300 MHz, $CDCl_3$) δ (ppm) 7.46 (1H, s, Im*H*), 7.05 (1H, s, Im*H*), 6.91 (1H, s, Im*H*), 3.92 (2H, t, ^{3}J = 7.2 Hz, C*H*$_2$), 1.81 (2H, m, C*H*$_2$), 1.21 (22H, m, C*H*$_2$), 0.88 (3H, t, ^{3}J = 7.0 Hz, C*H*$_3$); ESMS: *m/z* calc. for $[M + H]^+$ 293.30, found 293.30.

The synthesis and characterisation of **5** has been reported previously.[2]

Standard synthetic method for imidazole-stabilised NPs.

Chlorometallate anions were extracted into toluene from an aqueous solution using Aliquat 336 (1.1 eqv per negative charge on the anion). The layers were separated, and the imidazole ligand was added to the organic portion. This mixture was stirred in an ice bath for fifteen minutes before aqueous sodium borohydride (5 eqv) was administered dropwise. The reaction was stirred at room temperature for a further two hours before being washed with water and 1M HCl (aq). The aqueous washes were discarded, and the solution was filtered. Rotary evaporation gave the nanoparticulate product. Full TEM, ICP, and XPS analyses can be, found in the Digital Appendix.

AuNP.1$_{10}$

Standard synthesis, using $NaAuCl_4$ (0.10 g, 0.28 mmol), Aliquat 336 (0.12 g, 0.31 mmol), *N*-propyl-imidazole **1** (0.31 g, 2.8 mmol), $NaBH_4$ (0.06 g, 1.4 mmol), and toluene (25 ml). Significant agglomeration was observed, but some stable NPs were also present (0.021 g). UV-vis (toluene) PRB 532 nm; TEM mean diameter 18.2 nm (± 2.4, n = 39).

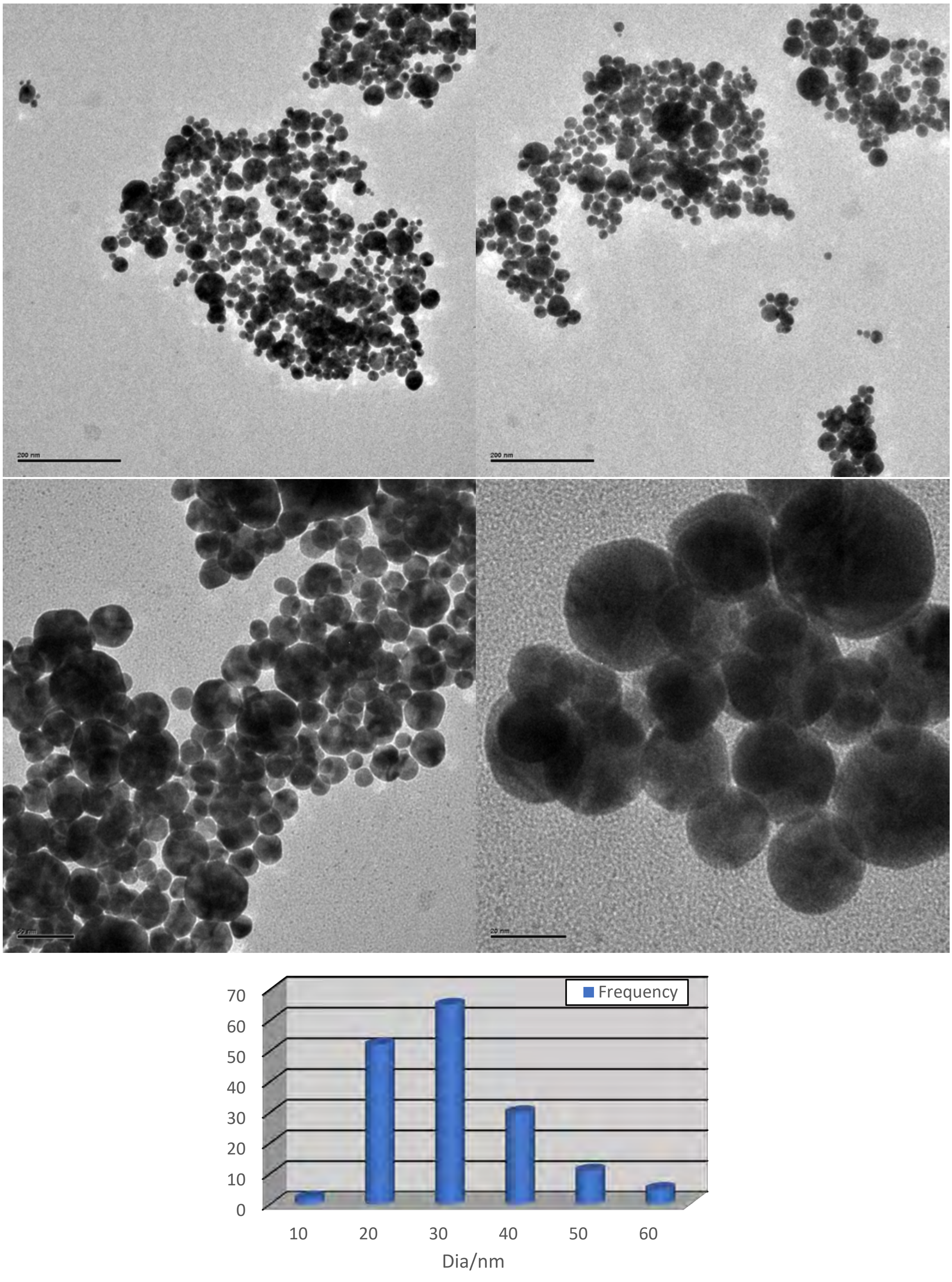

Figure S1. TEM images of **AuNP.1$_{10}$** and histogram of measured diameters.

AuNP.2$_1$

Standard synthesis, using $HAuCl_4$ (0.30 g, 0.89 mmol), Aliquat 336 (0.39 g, 0.97 mmol), *N*-hexyl-imidazole **2** (0.14 g, 0.89 mmol), $NaBH_4$ (0.17 g, 4.43 mmol), and toluene (25 ml), giving a thick purple oil (0.030 g). UV-vis (toluene) PRB 529 nm; TEM mean diameter 2.9 nm (± 1.7, n = 136).

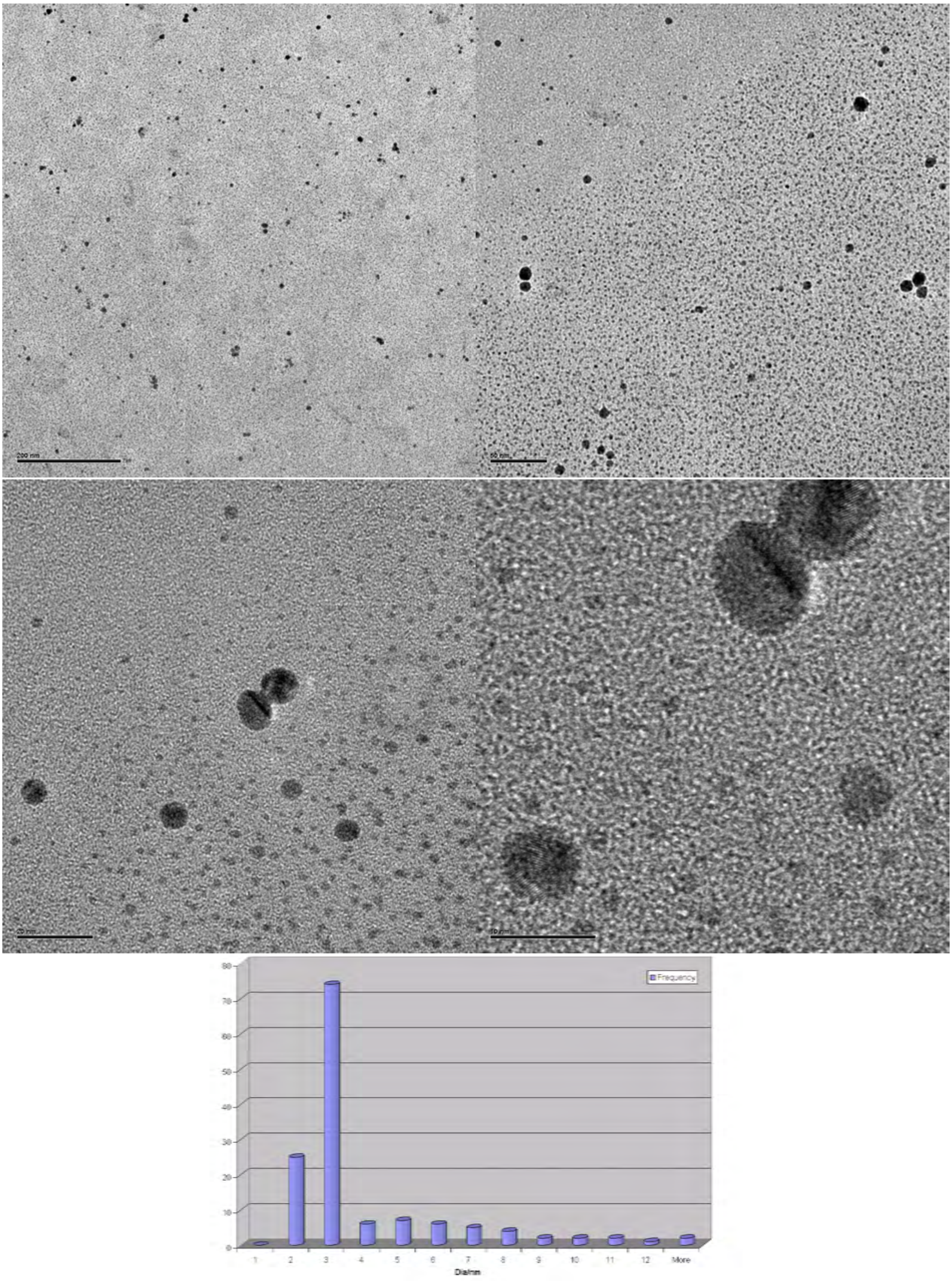

Figure S2. TEM images of **AuNP.2$_1$** and histogram of measured diameters.

AuNP.2$_{10}$

Standard synthesis, using $HAuCl_4$ (0.30 g, 0.89 mmol), Aliquat 336 (0.39 g, 0.97 mmol), *N*-hexyl-imidazole **2** (1.35 g, 8.86 mmol), $NaBH_4$ (0.17 g, 4.43 mmol), and toluene (25 ml), giving a thick purple oil (0.052 g). UV-vis (toluene) PRB 556 nm; TEM mean diameter 2.8 nm (± 1.1, n = 118).

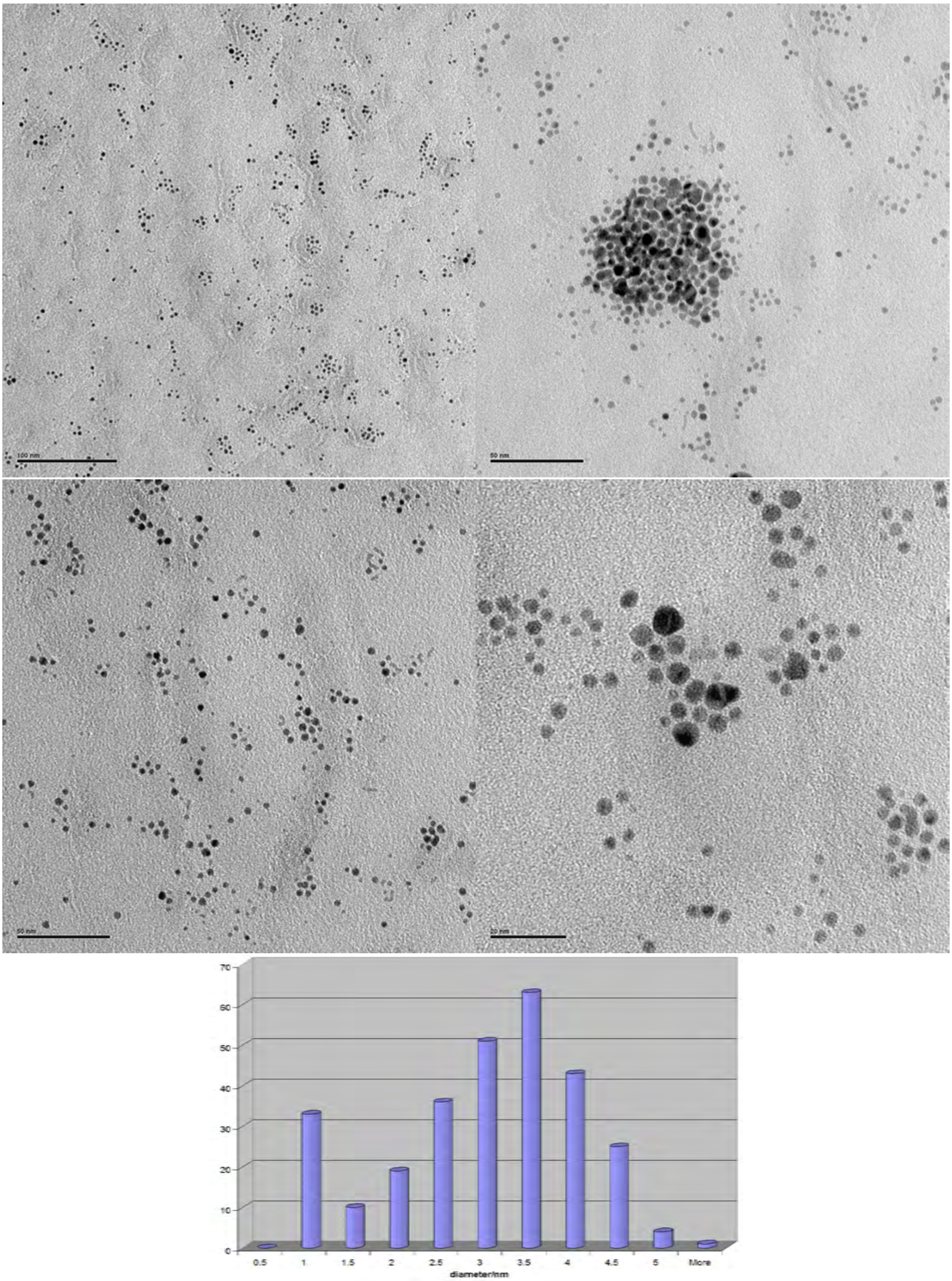

Figure S3. TEM images of **AuNP.2$_{10}$** and histogram of measured diameters.

AuNP.3$_1$

Standard synthesis, using $HAuCl_4$ (0.30 g, 0.89 mmol), Aliquat 336 (0.39 g, 0.97 mmol), *N*-dodecyl-imidazole **3** (0.21 g, 0.89 mmol), $NaBH_4$ (0.17 g, 4.43 mmol), and toluene (25 ml), giving a thick purple oil (0.042 g). UV-vis (toluene) PRB 522 nm; TEM mean diameter 3.9 nm (± 1.2, n = 285).

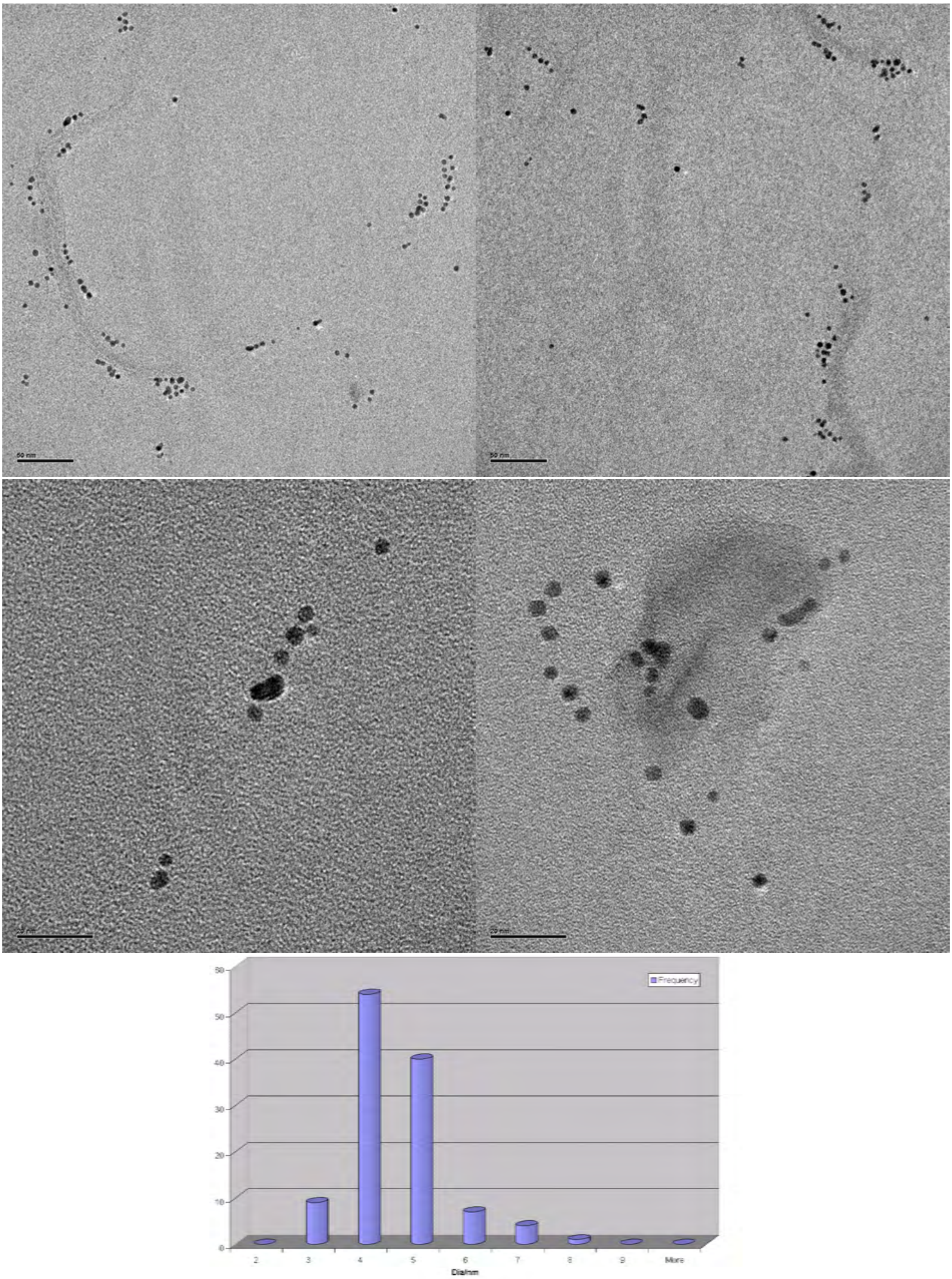

Figure S4. TEM images of **AuNP.3$_1$** and histogram of measured diameters.

AuNP.3$_{10}$

Standard synthesis, using $HAuCl_4$ (0.30 g, 0.89 mmol), Aliquat 336 (0.39 g, 0.97 mmol), *N*-dodecyl-imidazole **3** (2.10 g, 8.86 mmol), $NaBH_4$ (0.17 g, 4.43 mmol), and toluene (25 ml), giving a thick purple oil (0.063 g). UV-vis (toluene) PRB 520 nm; TEM mean diameter 2.3 nm (± 1.0, n = 398).

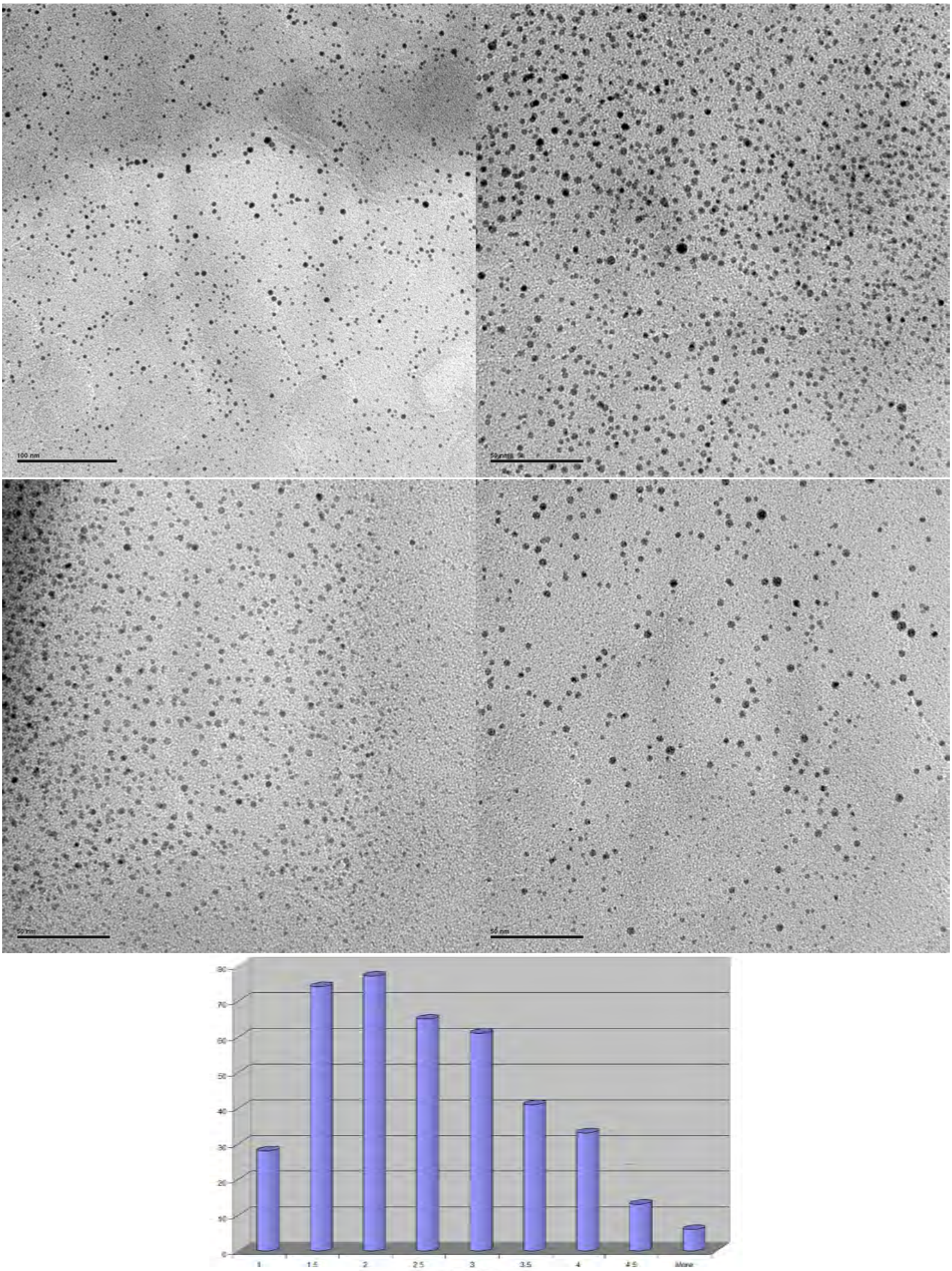

Figure S5. TEM images of **AuNP.3$_{10}$** and histogram of measured diameters.

PdNP.2$_1$

Standard synthesis, using K_2PdCl_4 (0.50 g, 1.95 mmol), Aliquat 336 (0.69 g, 2.15 mmol), *N*-hexyl-imidazole **2** (0.24 g, 1.95 mmol), $NaBH_4$ (0.17 g, 4.43 mmol), and toluene (25 ml), giving a thick brown-black oil (0.15 g). TEM mean diameter 1.9 nm (± 0.6, n = 484).

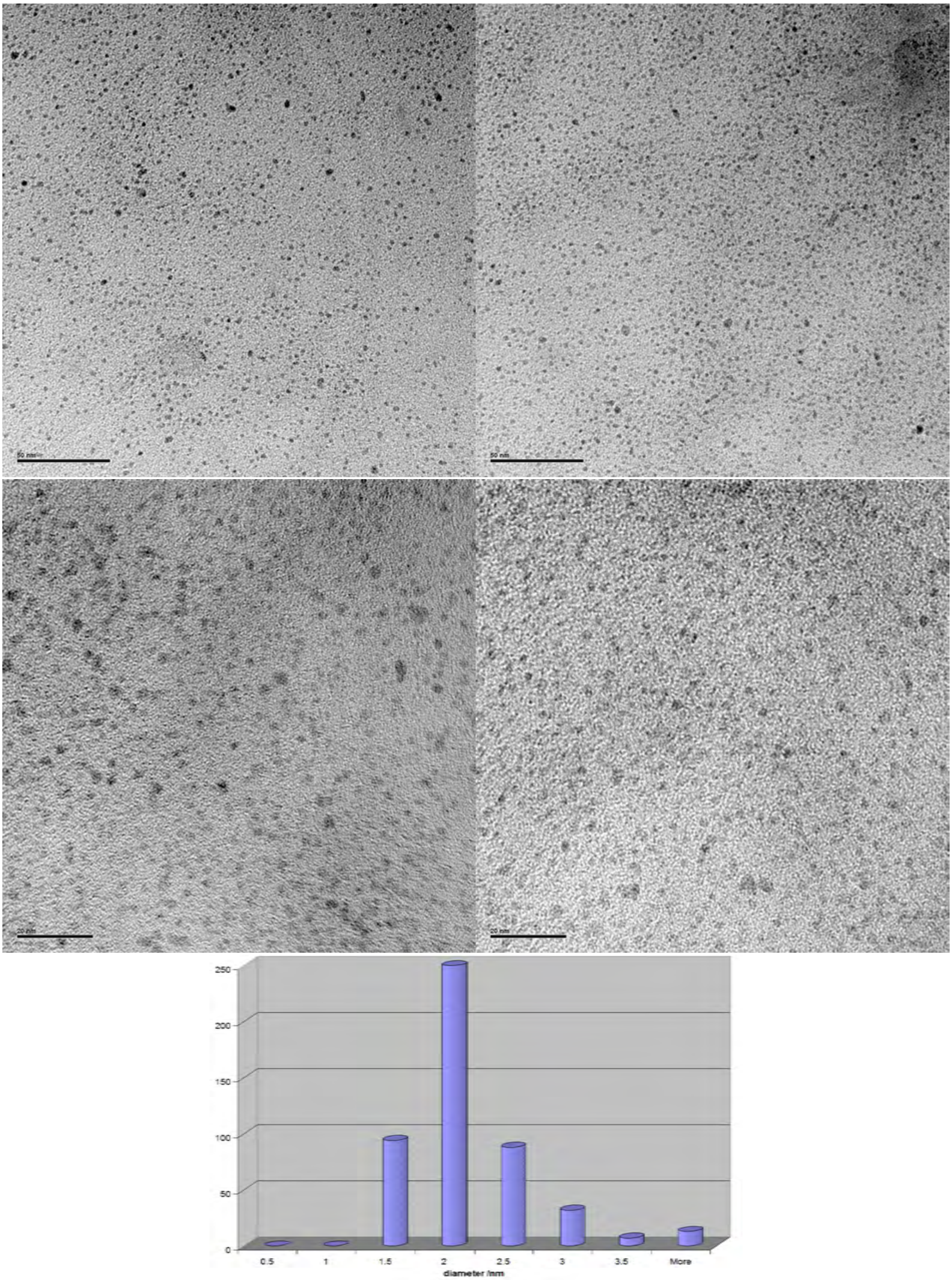

Figure S6. TEM images of **PdNP.2$_1$** and histogram of measured diameters.

PdNP.2$_{10}$

Standard synthesis, using K_2PdCl_4 (0.50 g, 1.95 mmol), Aliquat 336 (0.69 g, 2.15 mmol), *N*-hexyl-imidazole **2** (2.36 g, 19.5 mmol), $NaBH_4$ (0.17 g, 4.43 mmol), and toluene (25 ml), giving a thick brown-black oil (0.31 g). TEM mean diameter 1.6 nm (± 0.9, n = 130).

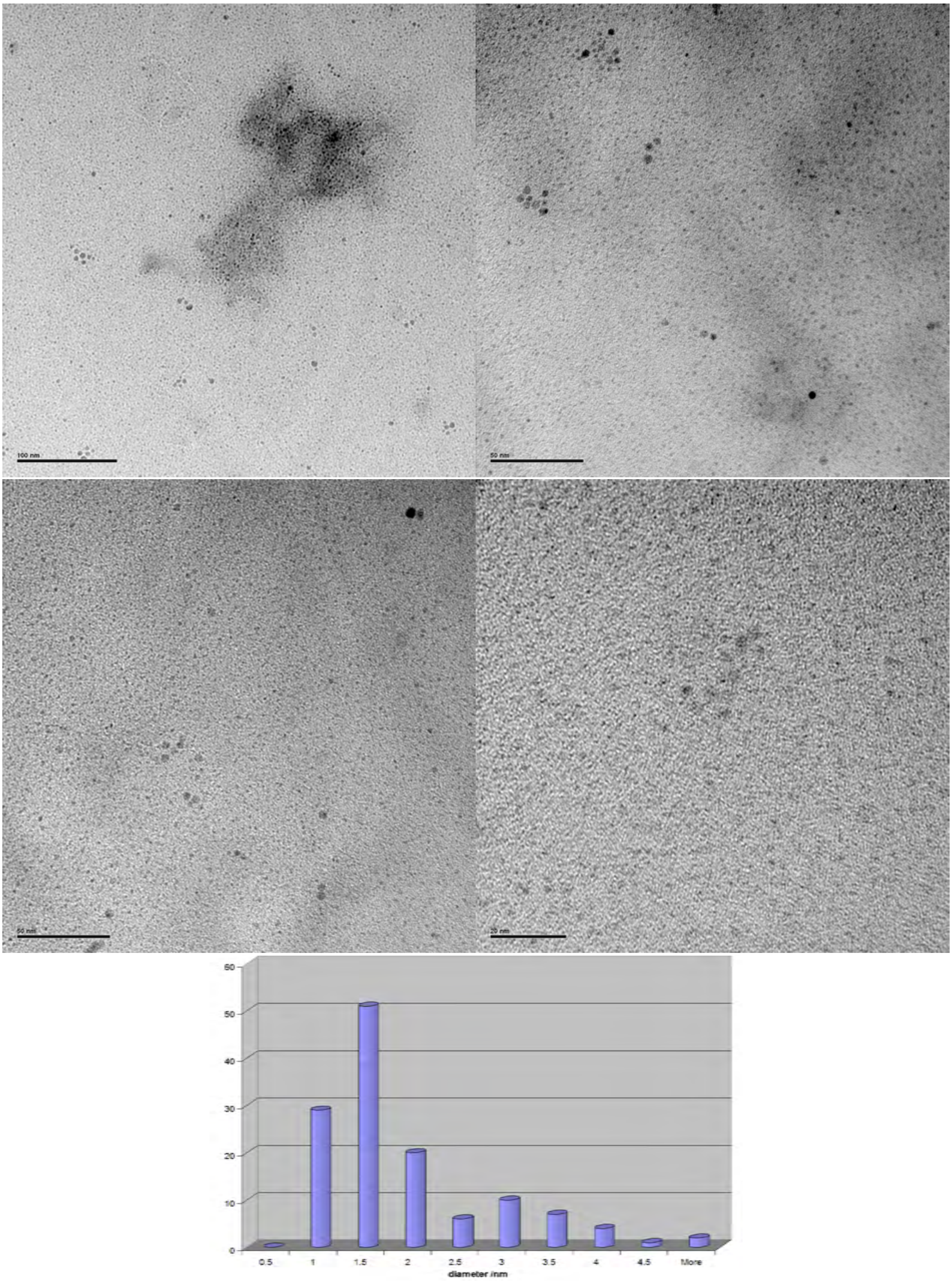

Figure S7. TEM images of **PdNP.2$_{10}$** and histogram of measured diameters.

PdNP.3$_1$

Standard synthesis, using K_2PdCl_4 (0.50 g, 1.95 mmol), Aliquat 336 (0.69 g, 2.15 mmol), *N*-dodecyl-imidazole **3** (0.37 g, 1.95 mmol), $NaBH_4$ (0.17 g, 4.43 mmol), and toluene (25 ml), giving a thick brown-black oil (0.19 g). TEM mean diameter 2.2 nm (± 0.9, n = 393).

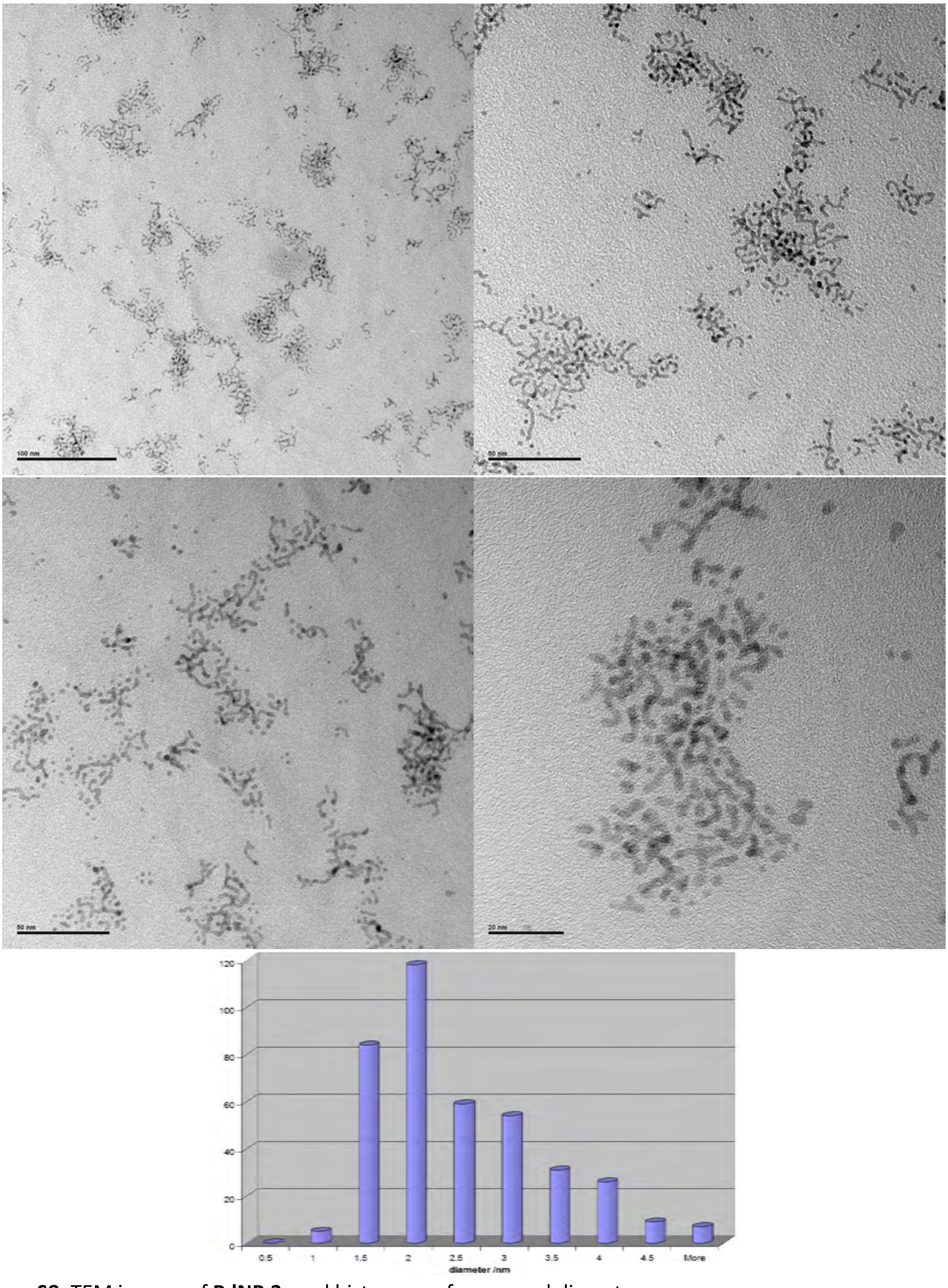

Figure S8. TEM images of **PdNP.3$_1$** and histogram of measured diameters.

PdNP.3$_{10}$

Standard synthesis, using K_2PdCl_4 (0.50 g, 1.95 mmol), Aliquat 336 (0.69 g, 2.15 mmol), *N*-dodecyl-imidazole **3** (3.67 g, 19.5 mmol), $NaBH_4$ (0.17 g, 4.43 mmol), and toluene (25 ml), giving a thick brown-black oil (0.32 g). TEM mean diameter 1.9 nm (± 0.2, n = 32).

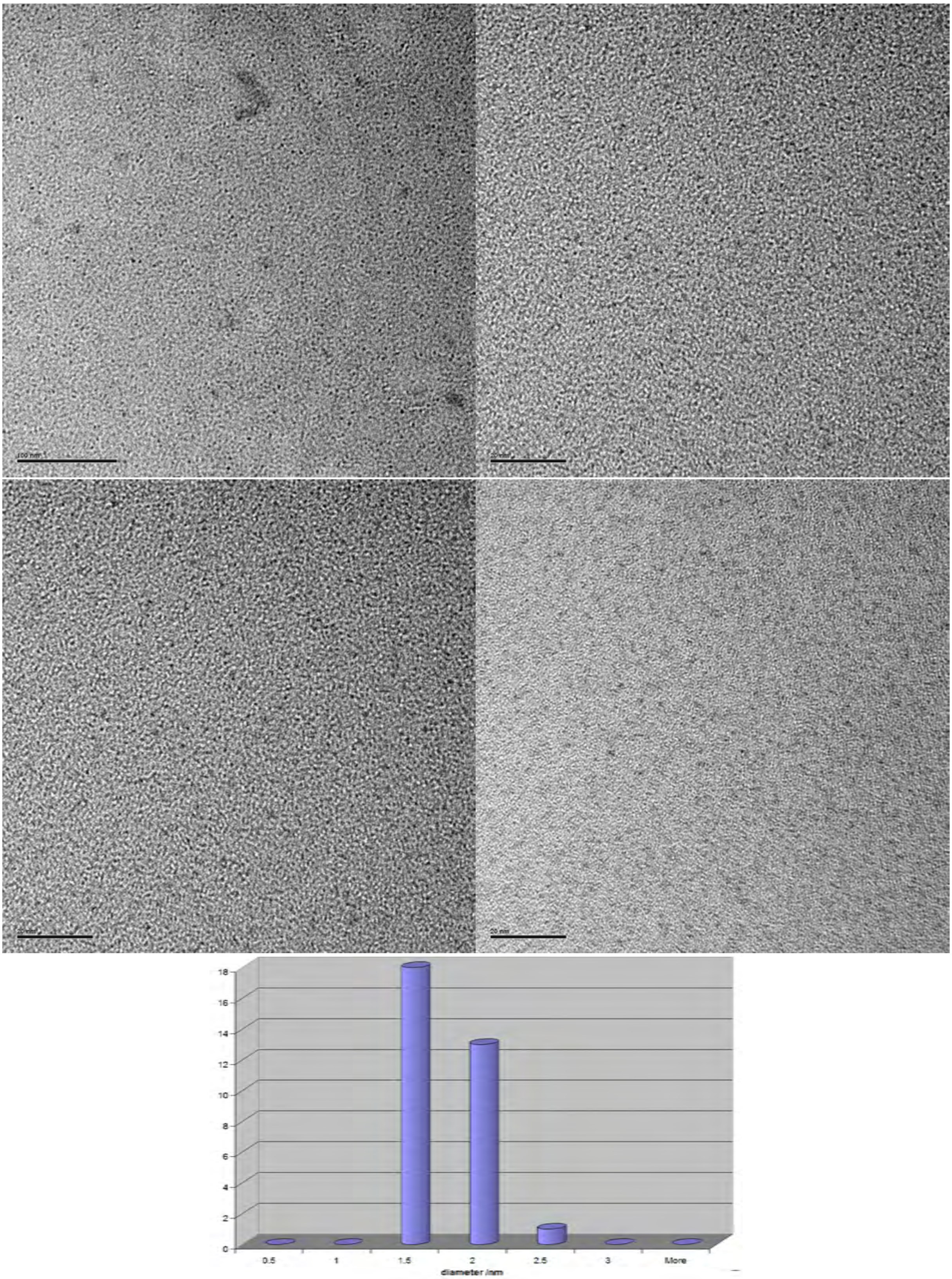

Figure S9. TEM images of **PdNP.3$_{10}$** and histogram of measured diameters.

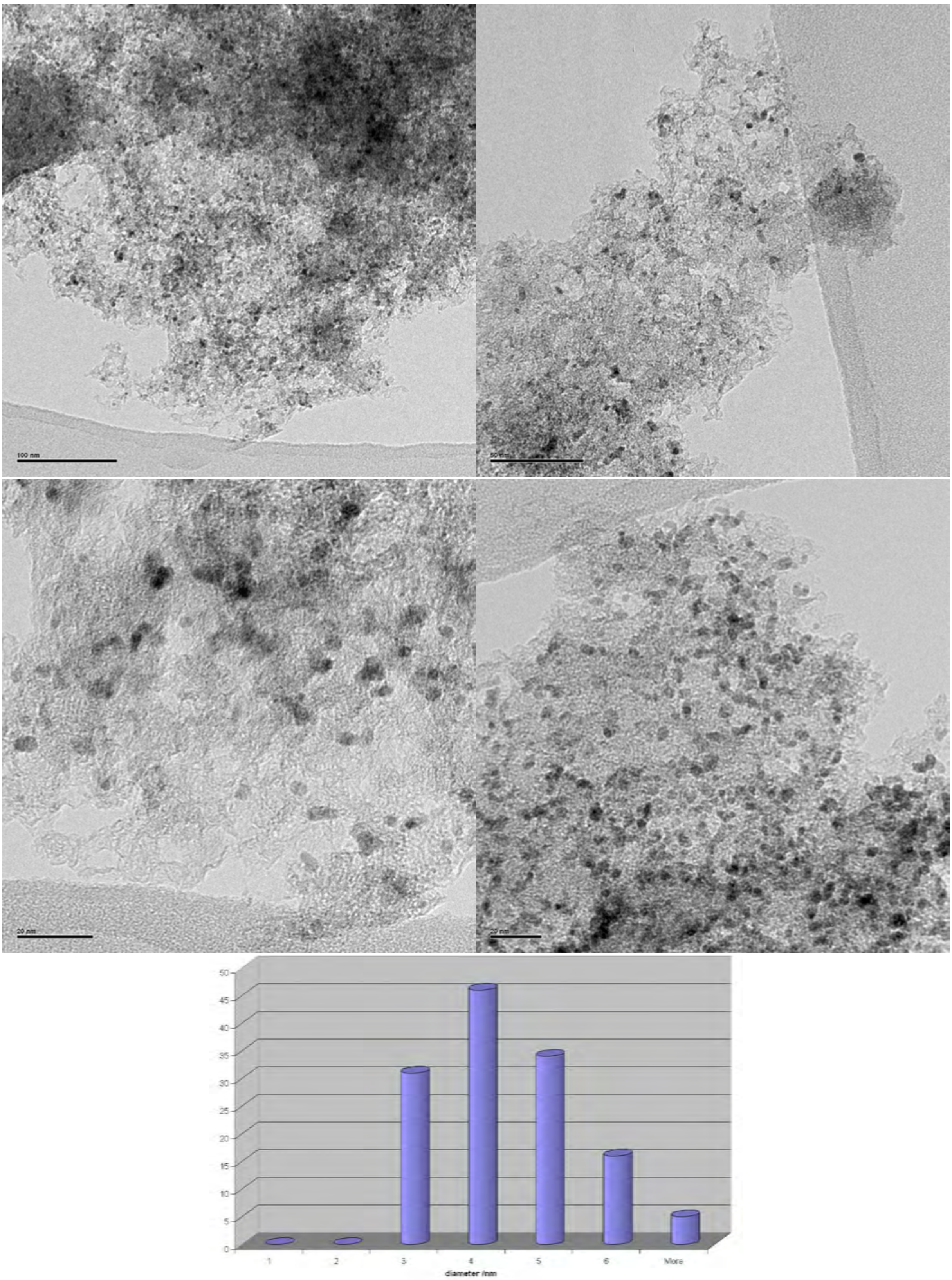

Figure S10. TEM images of **PdNP.3$_{10}$** after loading onto activated carbon (5 wt%) and histogram of measured diameters.

PtNP.2$_n$

Platinum acetoacetonate (0.10 g, 0.25 mmol) was dissolved in *N*-hexyl-imidazole **2** (2.26 g, 16.3 mmol) and heated at 200 °C for one hour with stirring, after which the solution had turned dark brown. Attempts to separate the NPs from the alkyl imidazole solvent using selective precipitation failed. TEM mean diameter < 1 nm (unable to measure).

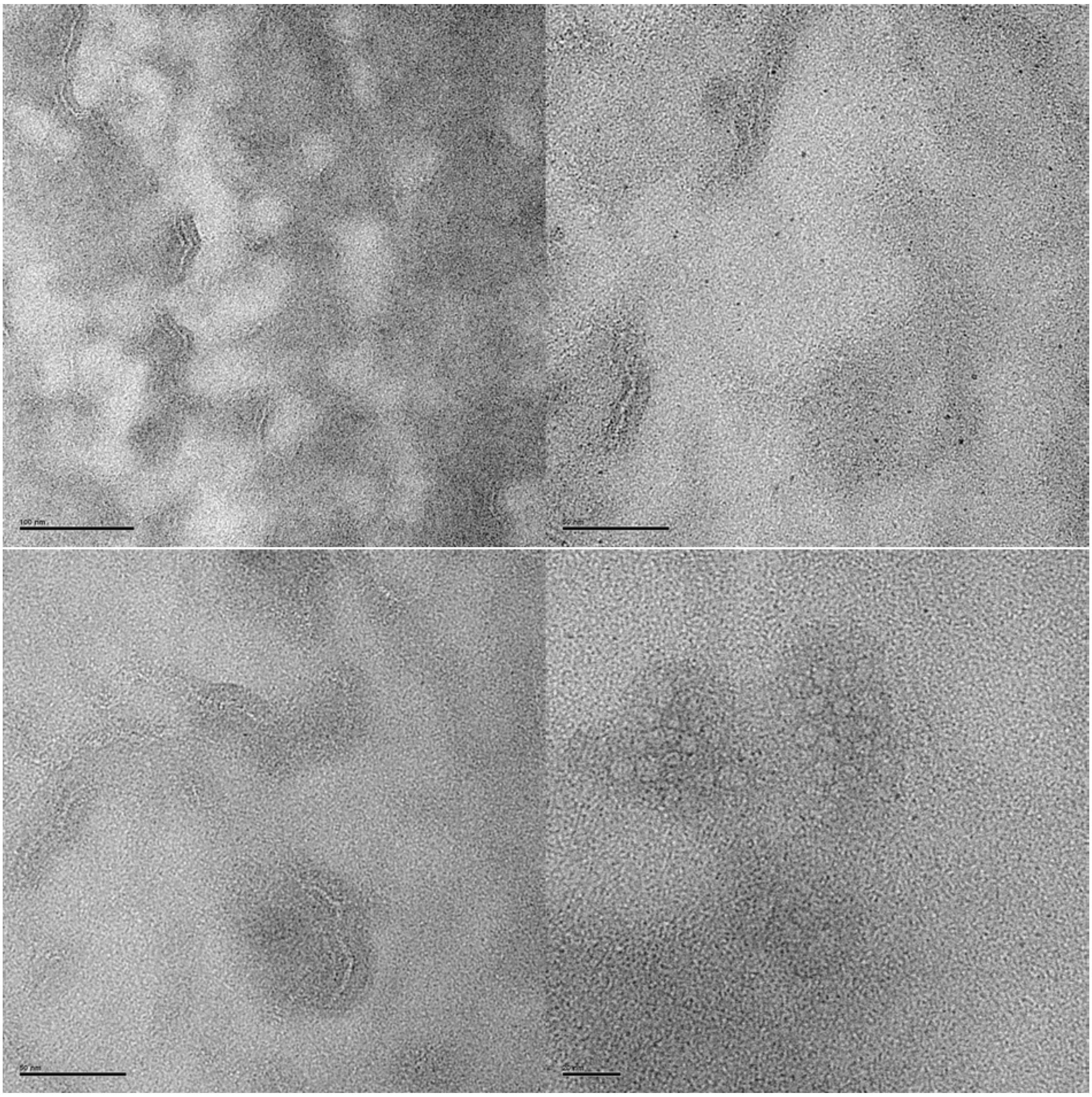

Figure S11. TEM images of **PtNP.2$_n$**.

AgNP.4_{10}

$AgNO_3$ (0.05 g, 0.30 mmol) was extracted from water (25 ml) into toluene (25 ml) using Aliquat 336 (0.60 g, 1.48 mmol). The aqueous layer was discarded. *N*-Hexadecyl-imidazole **4** (0.85 g, 3.0 mmol) was added to the toluene solution, which was cooled in an ice bath. After stirring for 15 min, $NaBH_4$ (0.06 g, 1.48 mmol) in water (5 ml) was administered dropwise, resulting in a red colouration. The reaction mixture was stirred at room temperature for 2 hr, after which the solvent was removed *in vacuo*. Addition of diethyl ether (25 ml) caused the separation of **AgNP.4_{10}** as a pink waxy solid (0.37 g), containing both Ag NPs and excess ligand. TEM mean diameter 25.6 nm (± 36.7, n = 29).

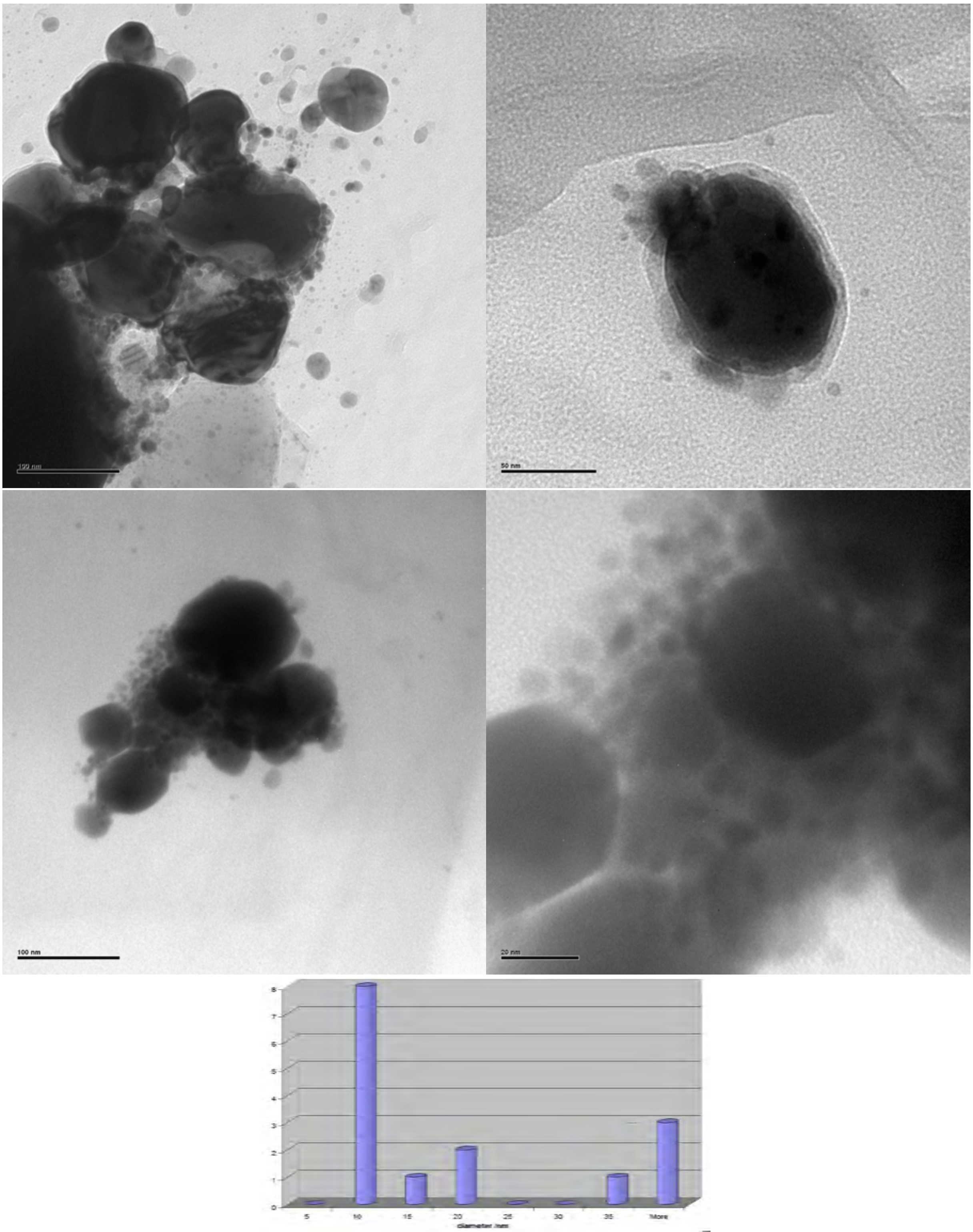

Figure S12. TEM images of **AgNP.4_{10}** and histogram of measured diameters.

AgNP.$5_{0.5}$

Compound **5** (0.10 g, 0.12 mmol) was added to a stirred solution of $AgNO_3$ (0.04 g, 0.24 mmol) in acetonitrile (50 ml), causing the formation of a fine precipitate. $NaBH_4$ (11.0 mg, 0.29 mmol) dissolved in MeOH (8 ml) was added dropwise over the course of a minute, causing the development of a brown colouration. The reaction mixture was stirred for an hour, after which the solvent was removed. The residue was triturated with water and saturated NH_4PF_6 (aq), and then collected by filtration. The solids were redissolved in acetonitrile, filtered, and subjected to rotary evaporation of the solvent giving **AgNP.$5_{0.5}$** as a brown solid (0.08 g). UV-vis (acetonitrile) PRB 419 nm; TEM mean diameter 11.1 nm (± 1.8, n = 85).

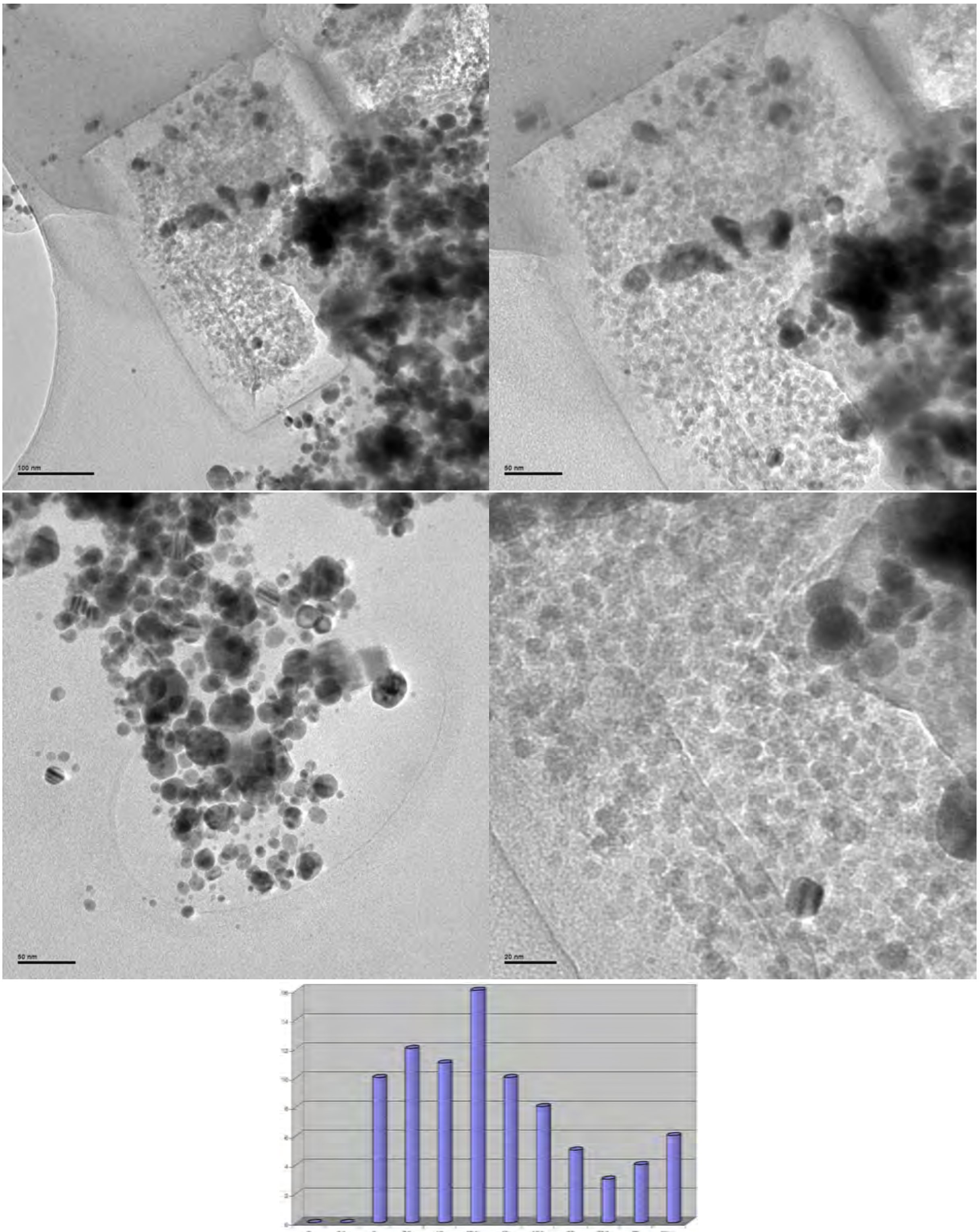

Figure S13. TEM images of **AgNP.$5_{0.5}$** and histogram of measured diameters.

AgNP.5$_{0.2}$

Prepared as for **AgNP.5$_{0.5}$**, using **5** (0.10 g, 0.12 mmol), $AgNO_3$ (0.10 g, 0.61 mmol), $NaBH_4$ (26.0 mg, 0.69 mmol), acetonitrile (50 ml), and MeOH (10 ml), giving **AgNP.5$_{0.2}$** as a brown solid (0.04 g). UV-vis (acetonitrile) PRB 413 nm; TEM mean diameter 7.5 nm (± 1.6, n = 154).

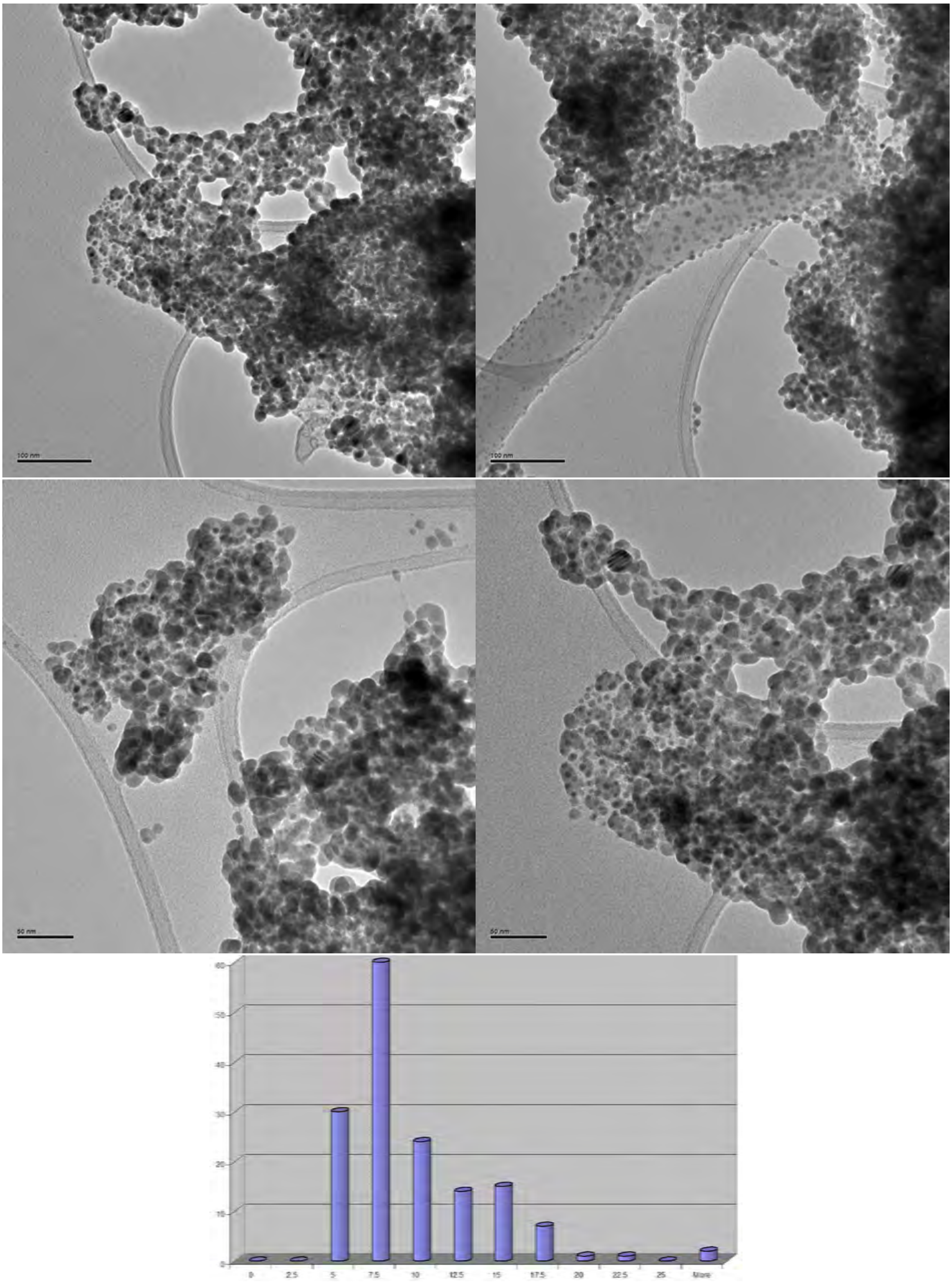

Figure S14. TEM images of **AgNP.5$_{0.2}$** and histogram of measured diameters.

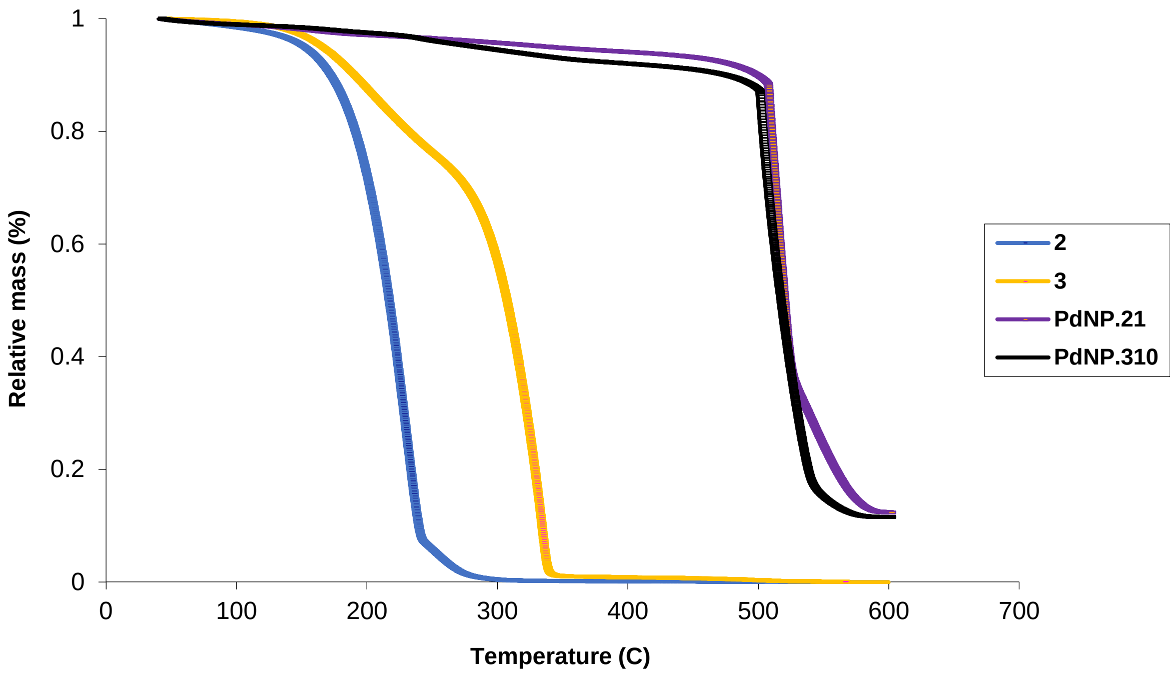

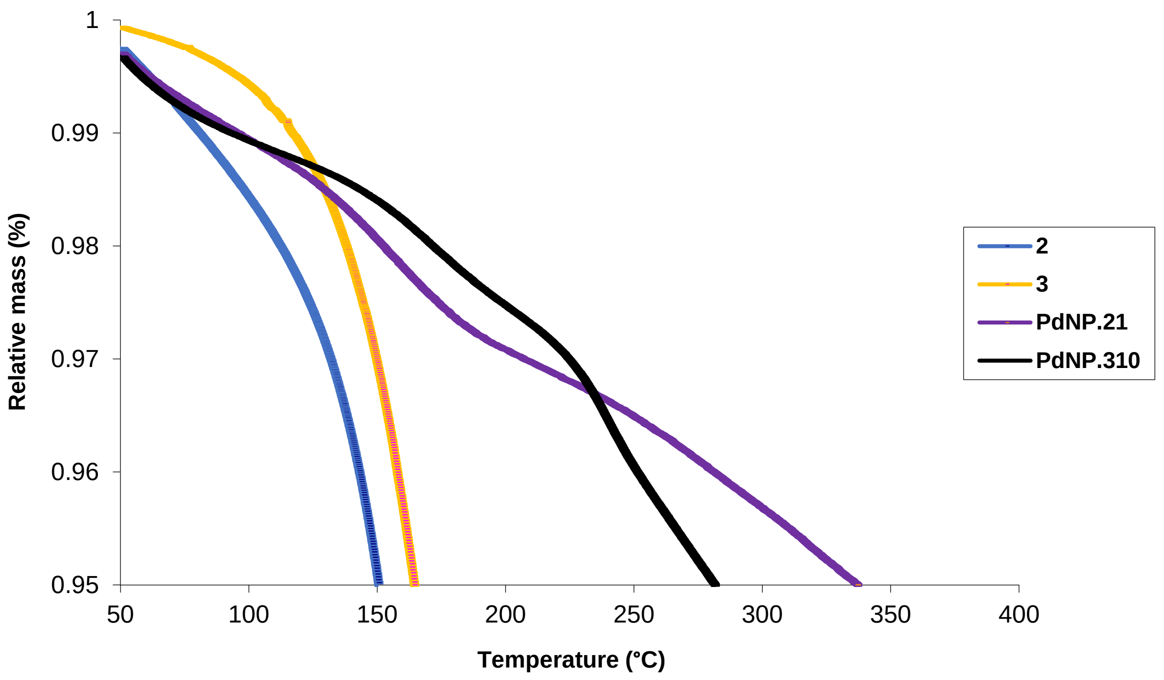

Figure S15. TGA traces for ligands **2** and **3**, and NP samples **PdNP.2$_1$** and **PdNP.3$_{10}$**. Lower panel shows details in 50 – 400 °C region.

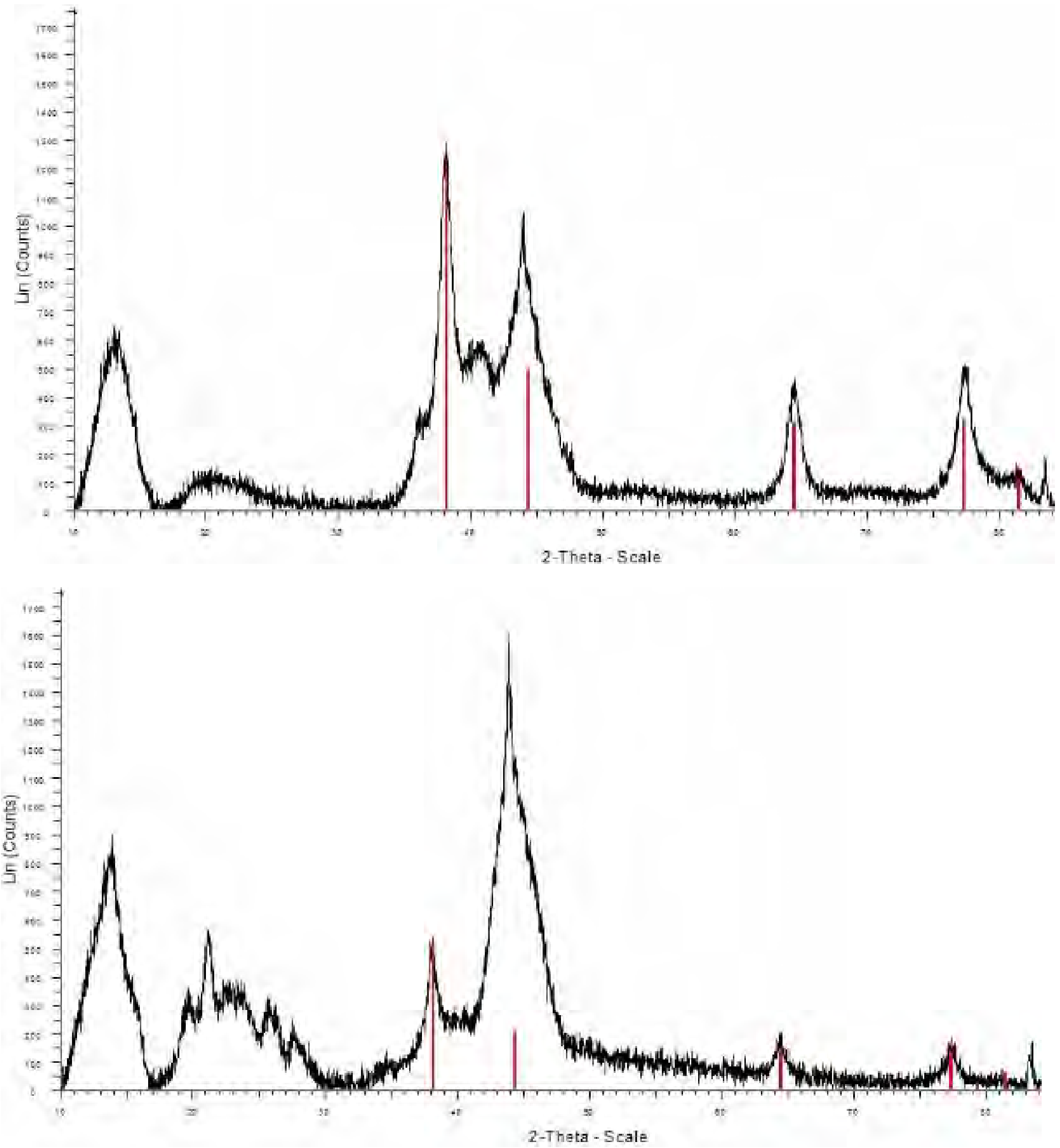

Figure S16. Powder x-ray diffraction patterns for **AgNP.5$_{0.5}$** (top) and **AgNP.5$_{0.2}$** (bottom). Red lines indicate the reflections seen in bulk elemental silver.

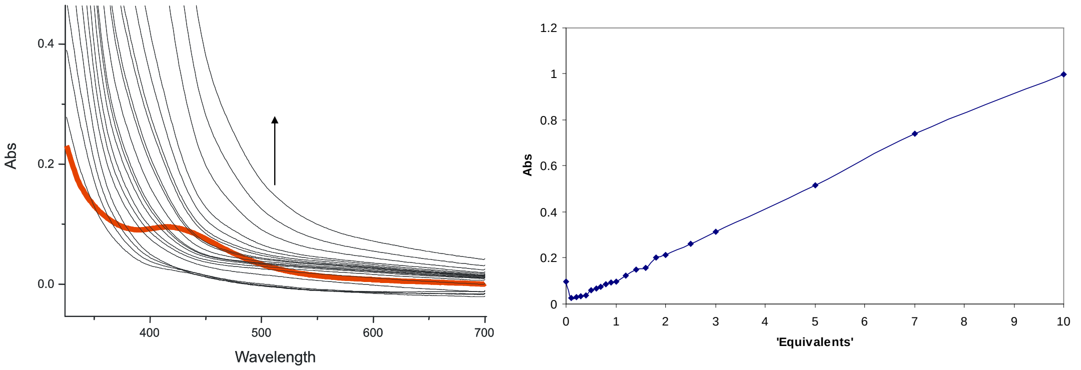

Figure S17. Effect of addition of TBA $AuCl_4$ to **AgNP.5$_{0.5}$** in 1:1 $MeCN/H_2O$ (293K). a) UV-visible spectra, with **AgNP.5$_{0.5}$** displayed in red. b) Absorbance at 413 nm plotted against nominal equivalents of TBA $AuCl_4$ added.

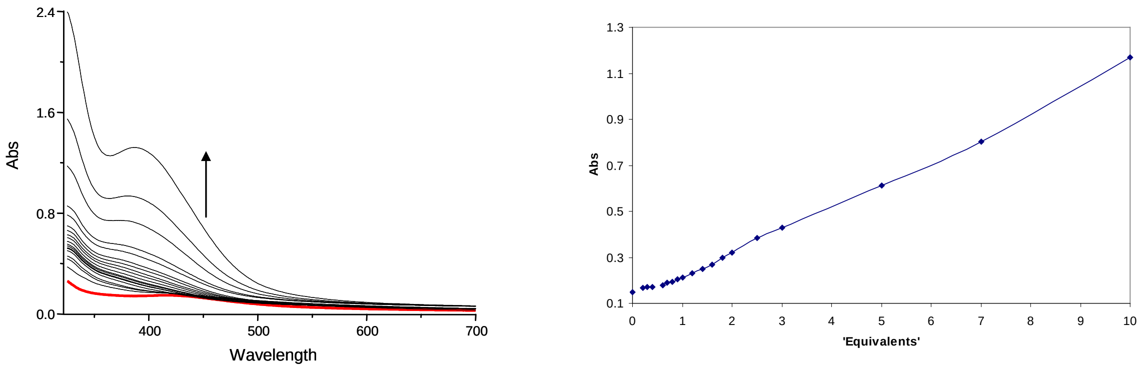

Figure S18. Effect of addition of TBA_2 Pd_2Cl_6 to **AgNP.5$_{0.5}$** in 1:1 $MeCN/H_2O$ (293K). a) UV-visible spectra, with 140 displayed in red. b) Absorbance at 413 nm plotted against nominal equivalents of TBA Pd_2Cl_6 added.

References

1 A. L. Patterson, *Phys. Rev.*, 1939, **56**, 978.

2 C. J. Serpell, J. Cookson, A. L. Thompson and P. D. Beer, *Chem. Sci.*, 2011, **2**, 494–500.
